# Effect of DA-9701 on Gastric Motor Function Assessed by Magnetic Resonance Imaging in Healthy Volunteers: A Randomized, Double-Blind, Placebo-Controlled Trial

**DOI:** 10.1371/journal.pone.0138927

**Published:** 2015-09-24

**Authors:** Yang Won Min, Byung-Hoon Min, Seonwoo Kim, Dongil Choi, Poong-Lyul Rhee

**Affiliations:** 1 Department of Medicine, Samsung Medical Center, Sungkyunkwan University School of Medicine, Seoul, Korea; 2 Biostatistics Team, Samsung Biomedical Research Institute, Seoul, Korea; 3 Radiology, Samsung Medical Center, Sungkyunkwan University School of Medicine, Seoul, Korea; University Hospital Llandough, UNITED KINGDOM

## Abstract

**Background:**

Improving gastric accommodation and gastric emptying is an attractive physiological treatment target in patients with functional dyspepsia (FD). We evaluated the effect of DA-9701, a new drug for FD, on gastric motor function after a meal in healthy volunteers using magnetic resonance imaging (MRI).

**Methods:**

Forty healthy volunteers were randomly allocated to receive either DA-9701 or placebo. After 5 days of treatment, subjects underwent gastric MRI (60 min before and 15, 30, 45, 60, 90, and 120 min after a liquid test meal). Gastric volume was measured through 3-dimensional reconstruction from MRI data. We analyzed 4 outcome variables including changes in total gastric volume (TGV), proximal TGV, and proximal to distal TGV ratio after a meal and gastric emptying rates after adjusting values at the pre-test meal.

**Results:**

Changes in TGV and proximal TGV after a meal did not differ between the DA-9701 and placebo groups (difference between groups -25.9 mL, 95% confidence interval [CI] -54.0 to 2.3 mL, *P* = 0.070 and -2.9 mL, 95% CI -30.3 to 24.5 mL, *P* = 0.832, respectively). However, pre-treatment with DA-9701 increased postprandial proximal to distal TGV ratio more than placebo (difference between groups 0.93, 95% CI 0.08 to 1.79, *P* = 0.034). In addition, pre-treatment with DA-9701 significantly increased gastric emptying as compared with placebo (mean difference between groups 3.41%, 95% CI 0.54% to 6.29%, *P* = 0.021, by mixed model for repeated measures).

**Conclusion:**

Our results suggested that DA-9701 enhances gastric emptying and does not significantly affect gastric accommodation in healthy volunteers. Further studies to confirm whether DA-9701 enhances these gastric motor functions in patients with FD are warranted.

**Trial Registration:**

ClinicalTrials.gov NCT02091635

## Introduction

Functional dyspepsia (FD) is a disorder characterized by chronic or recurrent upper abdominal pain or discomfort in the absence of a specific structural cause [1]. Although several mechanisms are suggested to underlie dyspeptic symptoms, disturbance in gastric accommodation and gastric emptying (GE) seem to be the major pathophysiological causes of FD. Impaired gastric accommodation to a meal and delayed GE are found in 40% and 30%-33.5% of patients with FD, respectively [2–4].

Various techniques have been used to evaluate gastric accommodation and emptying. Among them, gastric barostat is regarded as the gold standard to evaluate the accommodation response [5, 6]. However, this procedure is invasive and uncomfortable, limiting its feasibility in practice. Furthermore, the intragastric balloon appears to interfere with normal gastric physiology [7]. Although single photon emission computed tomography is a validated alternative non-invasive technique, it includes high exposure to ionizing radiation [8–10]. For measuring GE, scintigraphy has been used as the standard [3]. However, it also suffers from the disadvantage of exposure to radiation. In contrast, magnetic resonance imaging (MRI) is a non-invasive means of measuring gastric volume through three-dimensional (3-D) reconstruction. In addition, it has no interference with normal gastric physiology from an intragastric balloon, no risk of radiation, and is validated for assessing both gastric accommodation and emptying [11–15].

Improving gastric accommodation and emptying appears to be an attractive therapeutic target in patients with FD. Indeed, several drugs relaxing the gastric fundus and prokinetic drugs have been tried in clinical trials. However, most of them are not widely available due to adverse effects [16–19] or show disappointing effects for treating patients with FD [20–22]. Although acotiamide has recently shown efficacy on the symptom and gastric motor function in patients with FD, there are still unmet needs for the treatment of FD [23].

DA-9701 is a new drug for treating patients with FD that has been marketed in South Korea since 2011. In the phase III trial,[24] DA-9701 showed non-inferior efficacy to itopride in patients with FD. DA-9701 is formulated as a 50% ethanol extract from Corydalis Tuber and Pharbitidis Semen. These plants are in Oriental traditional medicine for the treatment of gastrointestinal (GI) disorders. DA-9701 has multiple mechanisms of action such as fundus relaxation, visceral analgesia, and prokinetic effects [25]. In animal studies, DA-9701 not only significantly enhances GE but also improves gastric accommodation [26–28]. Given the heterogeneous pathophysiological mechanisms of FD, DA-9701 with multiple action mechanisms, is a promising drug for patients with FD. However, effect of DA-9701 on gastric accommodation and emptying in humans has not been evaluated by an objective measurement. Thus, this study aimed to evaluate effect of DA-9701 on gastric accommodation and emptying after a meal in a group of healthy volunteers using 3-D gastric volume measurements by MRI.

## Materials and Methods

### Subjects

Healthy volunteers between 20 and 70 years of age without upper abdominal pain or discomfort and a structural abnormality on upper GI endoscopy performed within the preceding 6 months were eligible for the trial. The subjects were recruited at Samsung Medical Center, Seoul, Korea between 29th of July 2013 and 26th of September 2013. Before group allocation, the subjects underwent clinical history taking, physical examination, laboratory tests (complete blood count, serum chemistry profiles, urinalysis, and pregnancy test), 12-lead electrocardiogram, and upper GI endoscopy (if not done within the previous 6 months). Patients were excluded if they met any of the following criteria: (1) any functional GI disease or previous abdominal surgery; (2) diabetes mellitus under insulin or oral anti-hyperglycemic agent treatment; (3) significant cardiopulmonary diseases or any malignancies; (4) significant renal (serum creatinine level ≥ 1.5 × the upper normal limit) or liver disease (serum aspartate aminotransferase and alanine aminotransferase levels ≥ 2.5 × the upper normal limits; (5) taking medications that may alter gastric function within 2 weeks prior to the start of the study; (6) pregnancy or lactation; (7) females with inadequate contraception during the study period; (8) contraindications to MRI (e.g., cardiac pacemaker or metallic aneurysm clip); (9) allergic history to DA-9701; and (10) other conditions likely to interfere with study procedures, as judged by the investigator.

This study protocol was conducted in accordance with the Declaration of Helsinki and approved by the Institutional Review Board at Samsung Medical Center, Seoul, Korea on 15th of April 2013 (No. 2012-12-095). All subjects provided written informed consent before inclusion in the study. The study was registered at ClinicalTrials.gov (NCT02091635) after enrollment of participants started due to our delayed process. The authors confirm that all ongoing and related trials for this drug/intervention are registered.

### Study design

**Fig 1:**
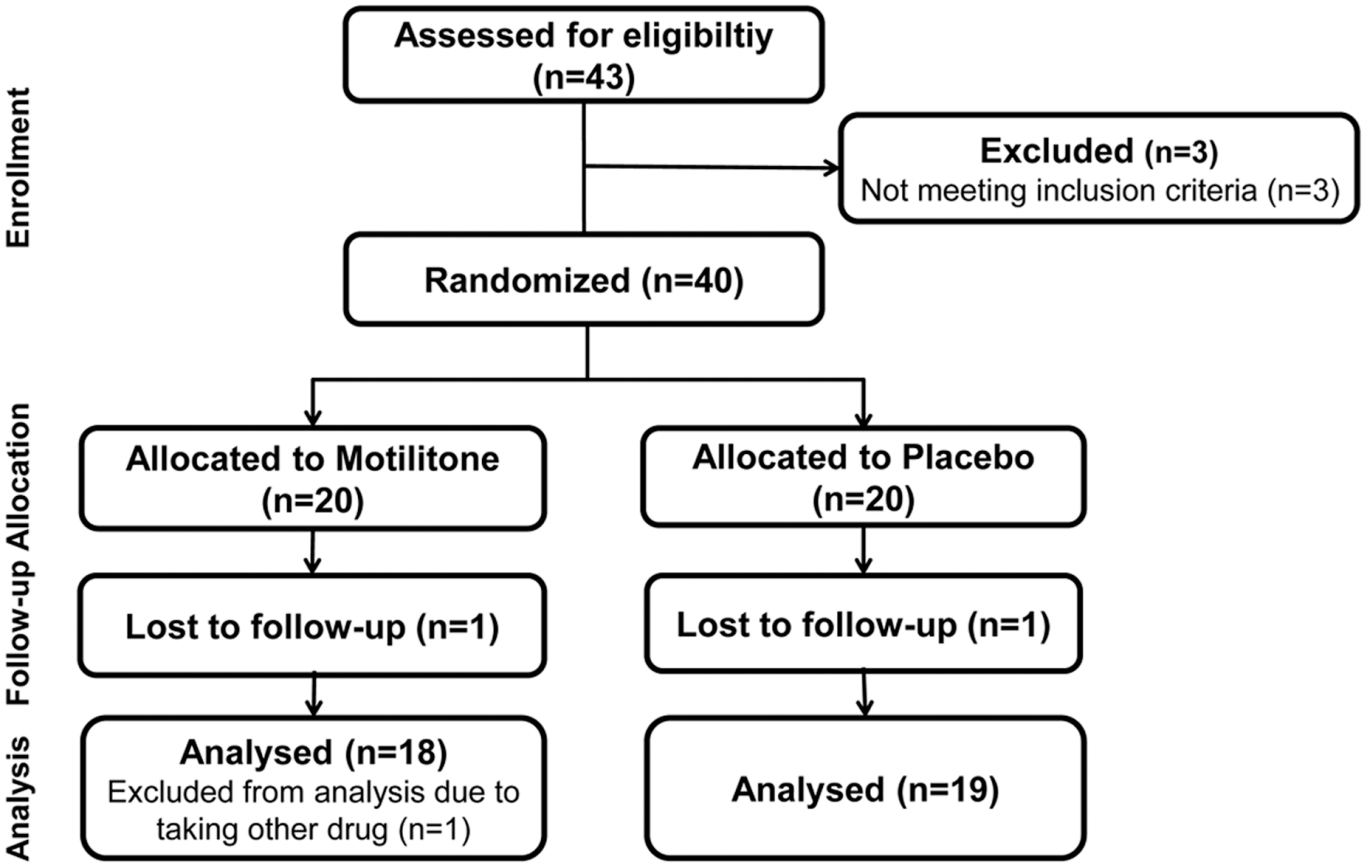
Flow sheet: enrollment, allocation, follow-up and analysis.

This was a randomized, double-blind, parallel group, placebo-controlled trial. Eligible subjects were randomly allocated in a 1: 1 ratio to receive either 60 mg (2 pills) of DA-9701 (Motilitone®, Dong-A ST, Yongin, Korea) (DA-9701 group) or placebo (placebo group) thrice daily (before meals) for 5 days (days 2–6). The randomization list employed a 1: 1 assignment ratio and a technique using a random permuted block design (Fig 1). The study drugs, DA-9701 and placebo, had the same weight, appearance, color, and texture and were packaged identically for the 2 groups. The drugs were identifiable only by randomization numbers and were provided by Dong-A ST.

**Fig 2:**
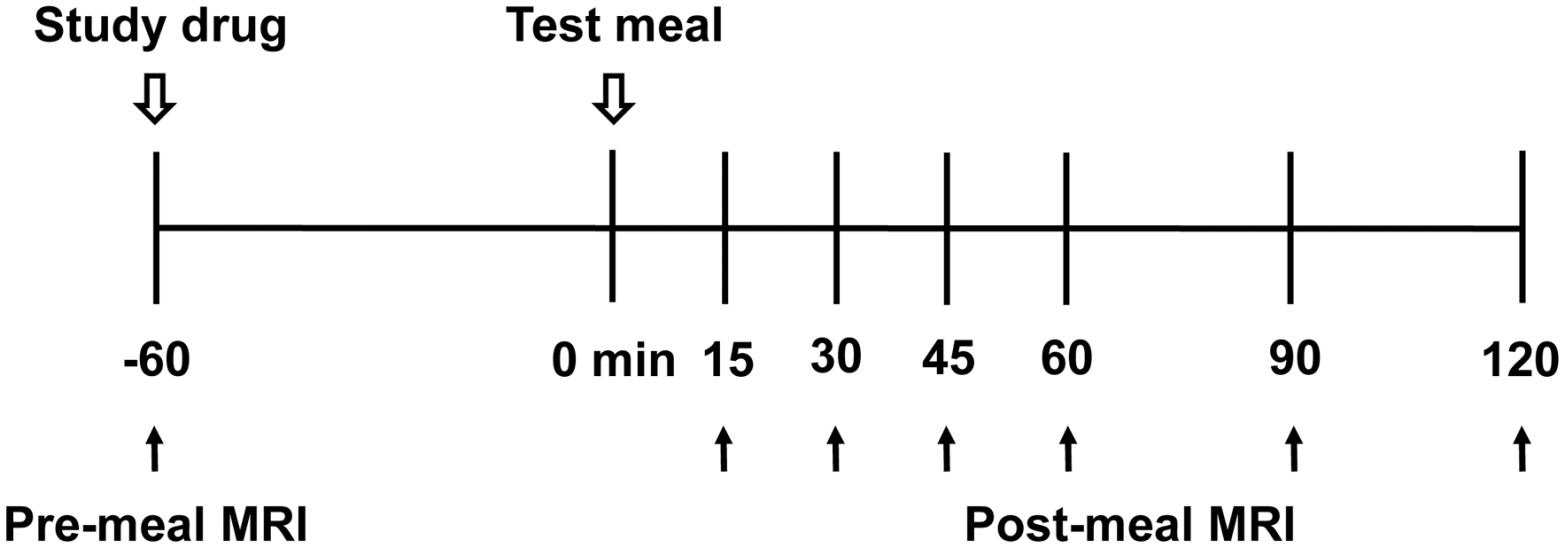
Study protocol on day 7.

At the end of the study (day 7), subjects underwent gastric MRI (pre-meal MRI) and took the study drug 60 min before administration of the test meal. Post-meal MRI was performed at 15, 30, 45, 60, 90, and 120 min after completing the test meal defined as time 0 min (Fig 2). The test meal consisted of 200 mL Nucare^®^ (200 kcal, carbohydrate: protein: fat = 57: 15: 27; Daesang, Seoul, Korea) and 200 mL water.

### MRI technique

**Fig 3:**
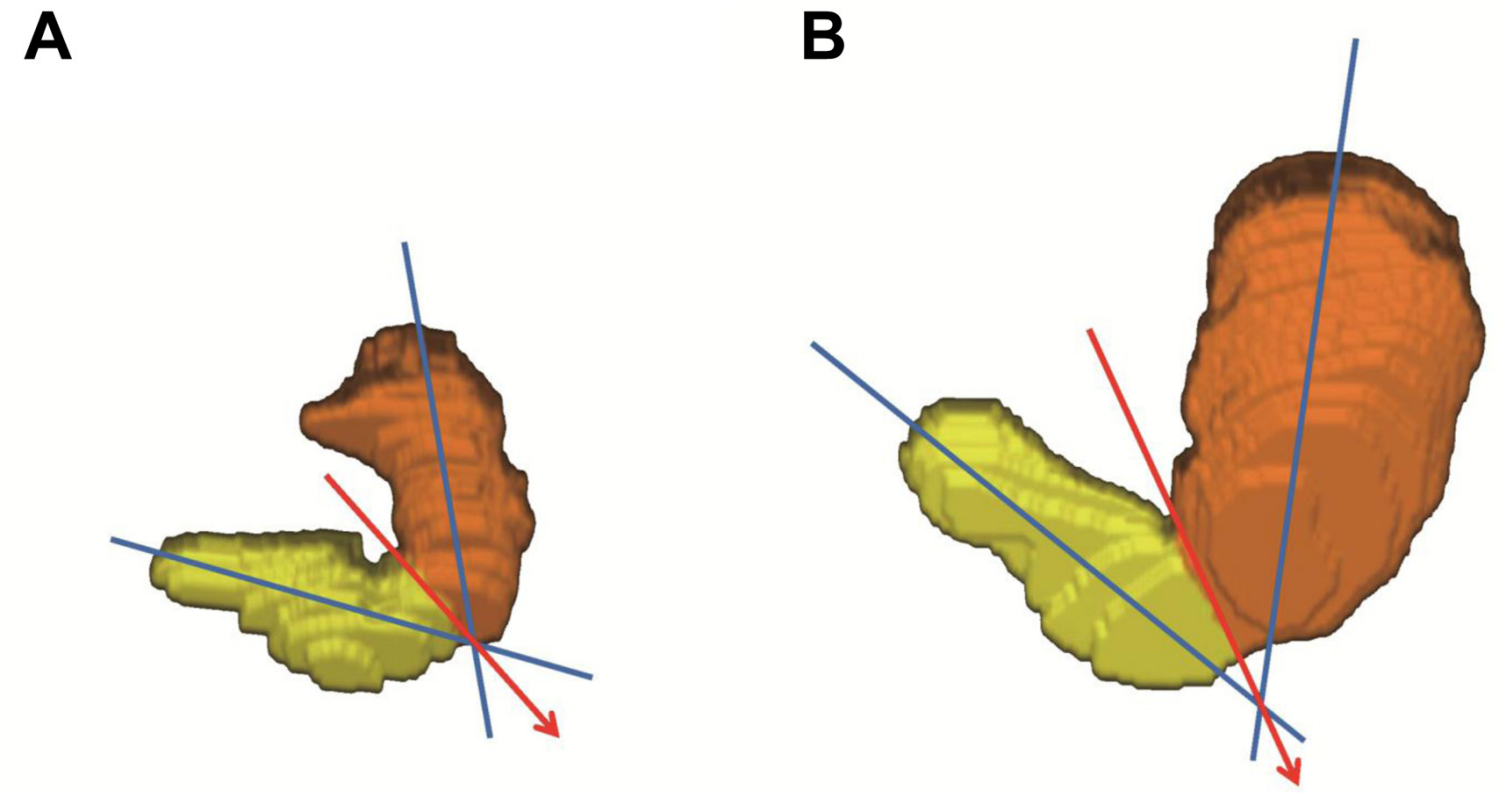
Three-dimensional visualization of stomach volumes measured by MRI before (A) and after (B) ingesting the test meal. The stomach was divided into the proximal (orange) and distal stomach (yellow) by an imaginary line (red arrow) drawn from the angular incisures to the point of contact between the 2 longitudinal axes (blue lines) through the center of the upper and lower parts of the stomach. The DA-9701 group case showed an increase in proximal to distal TGV ratio from 1.88 to 4.50 15 min after the test meal.

Gastric MRI was performed in the supine position using a 1.5T system (Interal Achieva, Philips Healthcare, Best, The Netherlands). Unenhanced images were obtained with turbo-field-echo sequence (repetition time, 4.1 ms; echo time, 1.63 ms; slice thickness 5 mm, no overlap, coronal). Thirty (or 35) consecutive slices covering the entire upper abdomen were obtained during 23 (or 25) seconds with one breath-hold (35 slices and 25 seconds were required in some subjects with a relatively large stomach). The areas of interest were drawn around gastric contents and air in each slice on the screen, which was identified by distinct contrast against the surrounding tissues, to determine the area of gastric contents and air. Obtained MRI data were transformed into 3-D images using AZE VirtualPlace^TM^ (AZE, Ltd., Tokyo, Japan), and gastric contents volume (GCV) was analyzed. Total gastric volume (TGV) was calculated by adding gastric air volume to GCV. The stomach was divided into the proximal and distal stomach using an imaginary line drawn from the angular incisures to the point of contact between the 2 longitudinal axes through the center of the upper and lower parts of the stomach (Fig 3). All areas of interest and imaginary lines dividing the stomach were drawn by co-author D.C. a qualified gastrointestinal radiologist, who was blinded to treatment group but not blinded to time-point and not blinded to patient.

### Assessment

#### Outcome variables

The primary outcome was change in TGV after the test meal, which was defined as difference between TGV 15 min after the test meal and at the pre-test meal. The secondary outcomes included GE rate (%) and changes in proximal TGV and proximal to distal TGV ratio after the test meal. Gastric emptying rates were calculated as follows: (GCV 15 min after the test meal-GCV at 30, 45, 60, 90, and 120 min after the test meal)/GCV 15 min after the test meal x 100 (%). Changes in proximal TGV and proximal to distal TGV ratio after the test meal were defined as difference between proximal TGV and proximal to distal TGV ratio 15 min after the test meal and at the pre-test meal. Proximal to distal TGV ratio was calculated as proximal TGV divided by the distal TGV.

#### Compliance and safety

The subjects returned their medication bottles and remaining drugs at the end of the study (day 7). Subjects were excluded if they did not take all of the medication. In addition, subjects were questioned with respect to adverse events and underwent laboratory tests and a 12-lead electrocardiogram.

### Statistical Analysis

Sample sizes were determined prospectively with reference to a previous study that used similar end points [29]. Sixteen subjects per group were required for a power of 80% under the 2-sided significance of 5% to detect a between-group difference of 50 mL and assuming SD of 50 mL of change in TGV after the test meal. To ensure inclusion of at least 16 subjects per group, 20 subjects per group were ultimately recruited to account for a potential withdrawal rate of 20%. The efficacy analysis was performed based on the modified intention-to-treat principle, which included all subjects who underwent randomization and who had endpoints that could be evaluated. Descriptive statistics for continuous variables and the categorical variables were presented as mean ± SD and frequency (proportion), respectively. The safety analysis set comprised all randomized subjects who received at least 1 study drug and provided a safety evaluation. Demographics in the 2 groups were compared using Fisher’s exact test for categorical data and the 2-sample *t*-test for continuous data. Gastric emptying rate was compared by repeated measures analyses with mixed model. Analysis of covariance model was used with the corresponding baseline value (values of measurement before the test meal) as the covariate to assess changes in TGV, proximal TGV and proximal to distal TGV ratio after the test meal to increase precision of the comparisons between the 2 groups by accounting for variation. Normality was checked before applying parametric tests. The least squares mean and 95% confidence intervals (CI) for between-group differences were calculated. Two-sided *P* values < 0.05 were taken as statistically significant. Statistical analyses were conducted using the SAS ver. 9.3 (SAS Institute, Cary, NC).

## Results

### Subjects

Forty-three healthy volunteers were screened. Of them 3 were excluded resulting in a total of 40 subjects who were randomly allocated. Two subjects (one each in the DA-9701 and placebo group) did not take all of the medication, which was not relevant to adverse events. One subject in the DA-9701 group was excluded because she took other drugs that may alter gastric function. Finally, 37 subjects were analyzed for efficacy in the current study. The subjects’ age was 34.4 ± 8.5 years (mean ± SD) and ranged from 22 to 56 years. Overall, 19 subjects (51.4%) were female. Baseline characteristics of the subjects did not differ between the 2 groups (Table 1). All continuous variables in Table 1 were normally distributed.

**Table 1:** Baseline characteristics of the subjects.

Characteristic	DA-9701 group (n = 18)	Placebo group (n = 19)	*P*-value*
**Age, years**			0.280
Mean ± SD	32.8 ± 7.6	35.8 ± 9.3	
Median (range)	30.5 (22.0–48.0)	35.0 (23.0–56.0)	
**Sex, n (%)**			1.000
Male	9 (50.0)	9 (47.4)	
Female	9 (50.0)	10 (52.6)	
**Height, cm**			0.658
Mean ± SD	166.8 ± 8.0	168.0 ± 8.6	
Median (range)	166.1 (155.7–184.4)	166.0 (153.1–184.4)	
**Weight, kg**			0.935
Mean ± SD	65.9 ± 12.4	65.6 ± 15.1	
Median (range)	64.3 (48.7–89.5)	63.9 (43.1–91.7)	
**Body mass index, kg/m** ^**2**^			0.581
Mean ± SD	23.6 ± 3.1	22.9 ± 3.5	
Median (range)	23.0 (19.9–30.0)	22.5 (17.1–28.5)	

* Fisher’s exact test for sex variable between DA-9701 and placebo groups and 2-sample *t*-test for continuous variables between DA-9701 and placebo groups after normality checking (*P* > 0.05)

### Effect of DA-9701 on change in total gastric volume after the test meal

Total gastric volume increased from 139.2 ± 41.1 mL to 486.7 ± 55.9 mL and from 151.9 ± 43.4 mL to 521.6 ± 45.7mL in the DA-9701 and the placebo group 15 min after the test meal, respectively. The SD of change in TGV was 44.7 mL in the DA-9701 group, which was similar to that assumed for sample size calculation. After adjusting values of TGV at the pre-test meal, difference in TGV between the 2 groups 15 min after the test meal was not statistically significant (−25.9 mL; 95% CI, −54.0 to 2.3 mL, *P* = 0.070; Table 2).

**Table 2:** Primary outcome. Changes in total gastric volume after ingesting a nutrient liquid meal (400 mL, 200 kcal) between the DA-9701 (n = 18) and the placebo group (n = 19).

Variables	DA-9701 group (n = 18)	Placebo group (n = 19)	^a^Mean difference (95% CI)	*P*-value
**TGV, mL (mean ± SD)**				
Pre-test meal	139.2 ± 41.1	151.9 ± 43.4		
15 min after test meal	486.7 ± 55.9	521.6 ± 45.7		
Change	347.5 ± 44.7	369.7 ± 40.9		
^b^ **Adjusted TGV at 15 min, mL (LSM ± SE)**	491.3 ± 9.9	517.2 ± 9.6	−25.9 (−54.0 to 2.3)	0.070

CI, confidence interval; TGV, total gastric volume; LSM, least squares mean

^a^ Difference in TGV between the DA-9701 and the placebo group 15 min after the test meal

^b^ Estimated from analysis of covariance adjusted for value of TGV at the pre-test meal after normality checking (*P* > 0.05)

### Effect of DA-9701 on gastric emptying

**Fig 4:**
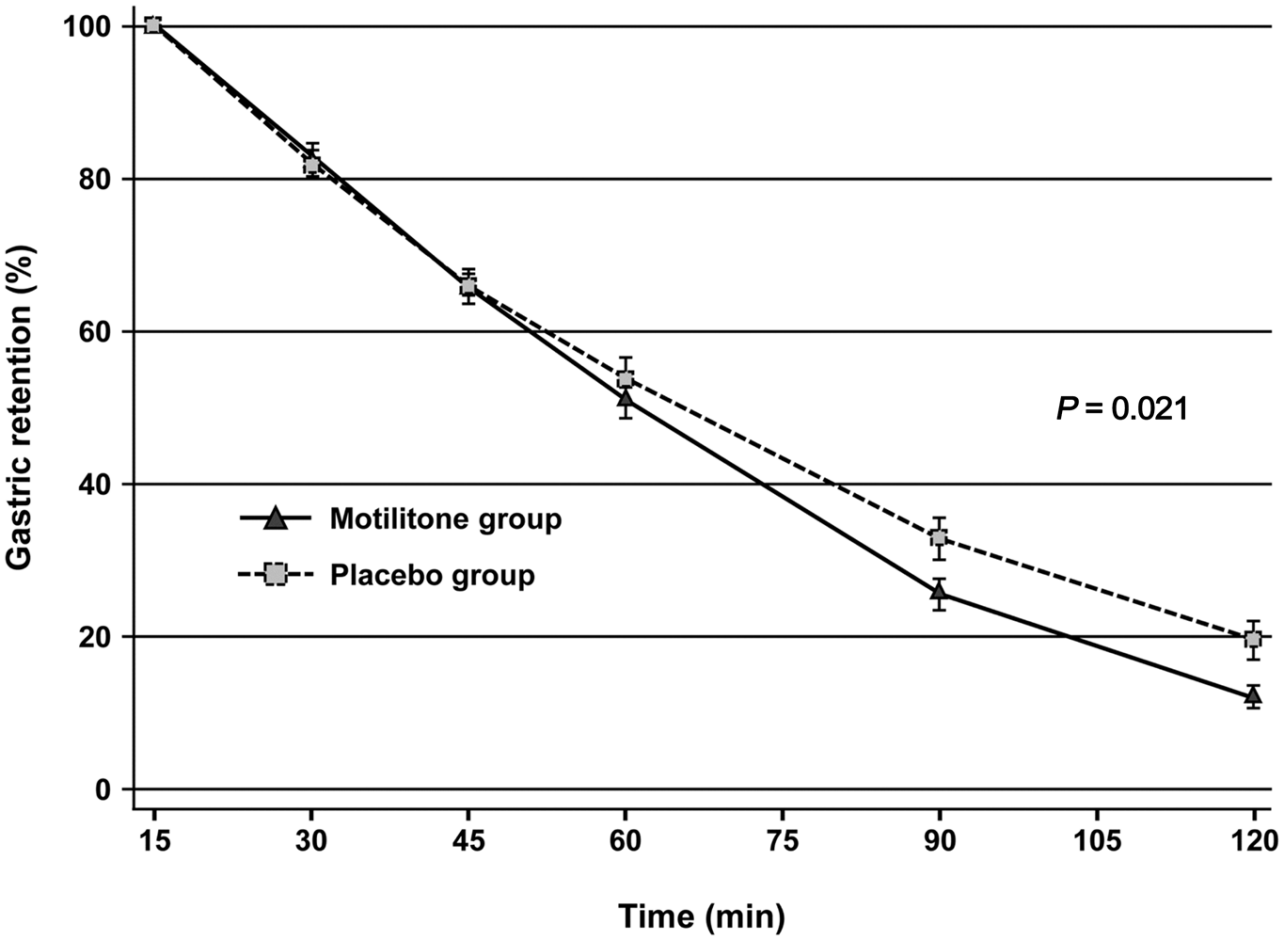
Gastric emptying after administration of the test meal (400 mL, 200 kcal) between the DA-9701 (n = 18) and the placebo group (n = 19). DA-9701 significantly enhanced gastric emptying (mean ± SE, *P* = 0.021, by mixed model for repeated measures).

Gastric emptying rates at 30, 45, 60, 90, and 120 min after the test meal were 17.2 ± 7.5%, 34.5 ± 8.3%, 49.0 ± 10.4%, 74.5 ± 8.9%, and 88.1 ± 6.0% in the DA-9701 group and 18.0 ± 7.1%, 34.1 ± 9.5%, 46.4 ± 12.4%, 67.3 ± 12.4%, and 80.6 ± 10.8% in the placebo group. Pre-treatment with DA-9701 significantly enhanced GE (difference between the 2 groups, 3.41; 95% CI, 0.54 to 6.29, *P* = 0.021 by mixed model for repeated measures; Fig 4).

### Effect of DA-9701 on change in proximal total gastric volume after the test meal

Proximal TGV increased from 90.6 ± 27.3 mL to 386.0 ± 43.2 mL and from 104.2 ± 32.0 mL to 396.4 ± 42.2 mL in the DA-9701 and the placebo group 15 min after the test meal, respectively. After adjusting values of proximal TGV at the pre-test meal, difference in proximal TGV 15 min after the test meal between the 2 groups was not significant (−2.9 mL; 95% CI, −30.3 to 24.5 mL, *P* = 0.832; Table 3).

**Table 3:** Secondary outcomes. Change in proximal total gastric volume and proximal to distal total gastric volume ratio after ingesting a liquid meal (400 mL, 200 kcal) among the DA-9701 group (n = 18) and the placebo group (n = 19).

Variables	DA-9701 group (n = 18)	Placebo group (n = 19)	^a^Mean difference (95% CI)	*P*-value
**Proximal TGV, mL**				
Pre-test meal	90.6 ± 27.3	104.2 ± 32.0		
15 min after test meal	386.0 ± 43.2	396.4 ± 42.2		
^b^ **Adjusted proximal TGV at 15 min, mL (LSM ± SE)**	389.9 ± 9.5	392.7 ± 9.3	−2.9 (−30.3 to 24.5)	0.832
**Proximal to distal TGV ratio**				
Pre-test meal	1.93 ± 0.40	2.33 ± 0.74		
15 min after test meal	4.14 ± 1.26	3.59 ± 1.38		
^c^ **Adjusted proximal to distal TGV ratio at 15 min, mL (LSM ± SE)**	4.33 ± 0.29	3.40 ± 0.29	0.93 (0.08 to 1.79)	0.034

CI, confidence interval; TGV, total gastric volume; LSM, least squares mean

^a^ Differences in proximal TGV and proximal to distal TGV ratio between the DA-9701 and the placebo group 15 min after the test meal

^b^ Estimated from analysis of covariance adjusted for value of proximal TGV at the pre-test meal after normality checking (*P* > 0.05)

^c^ Estimated from analysis of covariance adjusted for value of proximal to distal TGV ratio at the pre-test meal after normality checking (*P* > 0.05)

### Effect of DA-9701 on change in proximal to distal total gastric volume ratio after the test meal

Proximal to distal TGV ratio increased from 1.93 ± 0.40 to 4.14 ± 1.26 and from 2.33 ± 0.74 to 3.59 ±1.38 in the DA-9701 and the placebo group 15 min after the test meal, respectively. After adjusting values of proximal to distal TGV ratio at the pre-test meal, difference in proximal to distal TGV ratio 15 min after the test meal between the 2 groups was statistically significant (0.93; 95% CI, 0.08 to 1.79, *P* = 0.034; Table 3). Pre-treatment with DA-9701 increased proximal to distal TGV ratio after the test meal more than that with placebo.

### Safety results

Mild infectious colitis was reported during the treatment in the DA-9701 group (n = 1), which was considered unrelated with the study drug. However, the subject had taken other drugs that may alter gastric function and was excluded from the study. No other safety concerns were observed in either treatment group with regard to physical examinations, laboratory results, or electrocardiograms including QT interval.

## Discussion

Satisfactory therapeutic options are limited for patients with FD [30]. To date, very few drugs have proven efficacy [23], and adequate randomized controlled trials are lacking in patients with FD [20, 31–33]. Moreover, given the heterogeneous mechanisms of FD, several drugs with different mechanisms of action are required for the satisfactory treatment of patients with FD. In the present study, we evaluated the effect of DA-9701, a new drug for FD, on gastric accommodation and emptying after a meal. The gastric motor function was objectively evaluated using 3-D gastric volume measurements by MRI. Our results suggested that DA-9701 significantly increases proximal to distal TGV ratio after a meal and enhances GE rate in healthy volunteers.

Accommodation is an important mechanism of normal gastric physiology. It reduces gastric tone and increases compliance in response to a meal, allowing for an increase in proximal gastric volume without a corresponding rise in pressure [34, 35]. To evaluate gastric accommodation, we measured gastric volume change (TGV and proximal TGV) after a meal. Because impaired fundic accommodation to a meal facilitates redistribution of food to the antrum [36, 37], we also employed an additional index, proximal to distal TGV ratio to assess gastric accommodation. Gastric volumes were unaffected while proximal to distal TGV ratio increased in response to DA-9701. By definition, this study showed negative results in the primary outcome. However, these findings may be related to the study population and prokinetic effect of DA-9701. As gastric accommodation is not impaired in healthy volunteers, it might be difficult to increase gastric volumes by pre-treatment with DA-9701, but this would be different in patients with FD. In addition, the prokinetic effect of DA-9701 might reduce distal TGV more than proximal TGV by increasing antral contractility and contributing to the increase in proximal to distal TGV ratio over placebo without change in TGV and proximal TGV. Although proximal to distal TGV ratio is our secondary outcome for assessing gastric accommodation, this index remains to be validated. Therefore, the true efficacy of DA-9701 for gastric accommodation needs to be evaluated further in the group of patients.

In the present study, we used a nutrient liquid test meal. The liquid form of meal facilitated measuring the gastric content volume without contrast agent due to its distinct contrast against the surrounding tissues. Indeed, areas of interest could be easily drawn from the unenhanced images. As a liquid meal does not represent a standard meal, our results however need to be interpreted in the context of this limitation. In fact, liquids leave the stomach faster than solids, and normal emptying of liquids is frequently maintained even in severe gastroparesis for solids [38]. However, a significant difference in GE rate was detected with the use of DA-9701 in this study. In addition, we did not consider the volume of gastric secretions and its effect on the GE process when assessing GE rates [39]. Gastric secretion could affect liquid gastric emptying rate [40]. Although meal-induced gastric secretion would be similar between the 2 groups, there might be a possibility of different volume of secretion due to the drug. Effects of DA-9701 on gastric secretion are unknown. However, we believe that effects of drug on gastric function should be assessed without separating gastric secretion, which could show the real net effect and be practical.

As for duration of testing, we assessed GE up to 2 hr after the test meal. Although GE at 4 hr is optimal for assessing delayed GE, GE at 2 hr is also suitable for assessing rapid GE [41]. The current study involved healthy volunteers, and GE at 2 hr is appropriate to show the enhancing effect of DA-9701 on GE. In addition, gastric MRI was performed in the supine position. Human gastric motor function and relaxation volume does not differ between upright and supine positions [42]. Moreover, posture has only a minor impact on intragastric meal distribution and has no effect on GE [43]. Taken together, our results showed a true enhancing effect of DA-9701 on GE.

DA-9701 has an affinity for the D_2_, 5-HT_1A_, 5-HT_4_, and adrenergic α_2_ receptors. It has antagonistic effect on the D_2_ receptor and agonistic effects on the 5-HT_1A_, 5-HT_4_, and adrenergic α_2_ receptors [25]. DA-9701 enhances GE via D_2_ antagonism and 5-HT_4_ agonism [25]. Tetrahydroberberine (THB), isolated from Corydalis tuber, has micromolar affinity for the D_2_ and 5-HT_1A_ receptors. Oral administration of THB not only results in significantly accelerated GE but also restores delayed GE [44]. In addition, THB relaxes the proximal stomach via 5-HT_4_ agonism [44]. This coexistence of relaxation and contraction effects on the stomach might be explained by the regional differences of distribution and function of receptors in the stomach [45].

Gastric barostat is used as the standard for assessing accommodation response to a meal [5, 6]. In the current study, however, gastric MRI was employed to assess accommodation because gastric barostat is invasive and its intragastric balloon appears to interfere with normal gastric physiology [7]. Gastric MRI could also assess gastric emptying at the same time without risk of exposure to radiation, which is the disadvantage of scintigraphy, the current standard to evaluate gastric emptying. For these reasons, gastric MRI is a well validated and emerging means of assessing gastric motor functions [11–15]. The current study showed the potential of gastric MRI for gastric motor function assessments in a clinical setting.

Although the present study had some limitations including the liquid test meal and study population consisted of a group of healthy volunteers, we objectively demonstrated a significant enhancing effect of DA-9701 on gastric emptying using MRI. In conclusion, our results suggested that DA-9701 enhances gastric emptying and does not significantly affect gastric accommodation in healthy volunteers. Further studies are required to confirm whether DA-9701 enhances gastric accommodation and emptying in FD patients together with symptom evaluation.

## Supporting Information

S1 Checklist CONSORT Checklist. (DOCX)

S1 Dataset The dataset regarding gastric accommodation. (XLSX)

S2 Dataset The dataset regarding gastric emptying. (XLSX)

S1 Protocol The study protocol in Korean. (DOCX)

S2 Protocol The study protocol summarized in English. (DOCX)
