# Supplementary material for: Effect of DA-9701 on Gastric Motor Function Assessed by Magnetic Resonance Imaging in Healthy Volunteers: A Randomized, Double-Blind, Placebo-Controlled Trial
Source: PLoS One. 2015 Sep 24;10(9):e0138927. doi: 10.1371/journal.pone.0138927 (PMC4581730; doi:10.1371/journal.pone.0138927)
Supplement: S1 Protocol — (DOCX) [file pone.0138927.s004.docx]

임상시험계획서

Clinical Study Protocol

| **Study Title :** | **건강한 성인에서 위 적응에 대한 모티리톤(Motilitone)의 효과 연구: 자기 공명 영상을 통한 평가**  **Efficacy of Motilitone on gastric accommodation in healthy adult patients: Evaluation using MRI method** |
| --- | --- |
| **Protocol ID :** | **모티리톤** |
| **Protocol No. :** | **(Version 1.2)** |
| **Written Date :** | **2013년 06월 07일** |

| **임상시험 실시기관** | **삼성서울병원** |
| --- | --- |
| **시험책임자** | **소화기내과 이풍렬 교수** |

| **CONFIDENTIAL**  본 임상시험계획서에 포함된 정보는 (사)생명공익재단삼성서울병원의 자산으로 본 임상시험에 관여하지 않은 자는 삼성서울병원과 비밀 유지 계약을 체결한 경우가 아니면 본 임상시험계획서를 복사하거나 배포 받을 수 없습니다.  이 계획서를 제공 받은 분은 문서의 기밀 사항을 보장해 주시기 바랍니다. |
| --- |

# ❖ 임상시험계획서 개요

| **명칭** | 건강한 성인에서 위 적응에 대한 모티리톤(Motilitone)의 효과 연구 -자기 공명 영상을 통한 평가-  Efficacy of Motilitone on gastric accommodation in healthy adult patients: Evaluation using MRI method |
| --- | --- |
| **시험책임자** | 삼성서울병원 소화기내과 교수 이풍렬 |
| **공동연구자** | 삼성서울병원  소화기내과 교수 민병훈  영상의학과 교수 최동일  삼성생명과학연구소 통계지원팀 김선우 박사  소화기내과 임상전임강사 민양원 |
| **실시기관** | 삼성서울병원 / 서울 강남구 일원동 50 |
| **후원기관**  **모니터** | 동아제약주식회사ㅇ  삼성서울병원 A-CRO팀 |
| **목적** | 1차 목적.  건강한 성인에서 위 적응에 대한 Motilitone의 효과를 MRI를 통하여 평가하고자 한다  2차 목적.   1. 건강한 성인에서 위배출율에 대한 Motilitone의 효과를 MRI를 통하여 평가하고자 한다 2. Motilitone의 안전성 평가 |
| **시험설계** | 단일기관, 연구자임상시험, 이중눈가림, 위약대조, 무작위배정, 평행, 비교 시험 |
| **피험자 수** | **총 피험자 수: 40명 (탈락율 20% 적용)**   \| **분류** \| **건강한 피험자** \| \| \| \| --- \| --- \| --- \| --- \| \| **시험군**  **(Motilitone 180mg복용군)** \| **대조군**  **(위약 복용군)** \| **총 수** \| \| **유효성 평가례 수** \| 16명 \| 16명 \| 32명 \| \| **탈락율 20% 포함한 수** \| 20명 \| 20명 \| 40명 \|   피험자 수 산정은 기존 문헌에 근거하여 (ref: Neurogastroenterol Motil. 2009; 21: 697-e37^14)^) 일차유효성변수의 표준편차를 50 mL로 가정하고, 실험군과 대조군간 일차변수가 최소한 50 mL 이상 차이남을 기대할 때, 유의수준 5%, 검정력 80%하에서 20% 탈락율을 예상하면 각 군당 20명이 필요하다. |
| **대상 피험자** | 위장관계 증상이 없는 건강한 성인 |
| **피험자의  선정기준** | **<선정 기준>**   1. 만 20세 이상 만 70세 미만의 성인 2. 상복부 통증 혹은 불편감에 대해 무증상인 자 3. 본 시험에 참여할 것을 자발적으로 서면 동의한 자 4. 최근 6개월 이내 내시경 검사상 기질적 병변이 없는 자   **<제외 기준>**   1. 다른 기능성 위장 질환의 증상을 함께 보이는 환자 2. 임신 혹은 수유 중인 환자 3. 복부 수술의 기왕력이 있는 환자 4. 당뇨 환자 5. 심각한 다른 기저 질환이 있는 환자 6. 사용되는 약물에 알레르기가 있는 경우 7. 위장 운동 촉진제나 제산제 등을 복용하고 있는 환자는 최소 2주 전에 중단하여야 한다. 8. 간기능장애 (AST/ALT 수치가 정상 상한치의 2.5배 이상)가 있는 자 9. 신기능장애 (Serum creatinine이 정상 상한치의 1.5배 이상)가 있는 자 10. 아래와 같은 MRI검사를 받을 수 없는 환자   1) 심장박동기(Cardiac pacemaker)를 시술한 분  2) 동맥류협자(metallic aneurysm clip)를 시술한 분  3) 신경자극기(neurostimulator)를 시술한 분  4) 달팽이관 이식(cochlear implant)를 시술한 분  5) 임신중이거나 임신이 의심되는 분   1. 가임기 여성이면서 피임에 동의하지 않은 자 2. 기타 위 항목들 외에 연구자가 시험 참여에 부적합하다고 판단한 경우 |
| **임상시험용 의약품** | 시험약: Motilitone 180 mg(30mg/정)정제 또는 해당 위약 정제 |
| **시험 방법** | 스크리닝을 통과한 피험자는 MRI를 측정한다.  **MRI 측정방법:**  Test meal로 뉴케어200ml(200kcal, carbohydrate: protein: fat =57: 15: 27 )를 물 200 mL와 혼합한 후 섭취하도록 한다. 섭취 전, 섭취 후 15분, 30분, 45분, 60분 90분, 120분에 (총 7회) 촬영을 시행 한다. |
| **투여방법  및 투여기간** | Motilitone 1회 2정씩(30mg 2정) 또는 위약을 1일 3회 식전에 경구 투여하며, 총 5일 복용 후 Pre meal MRI 측정 직후에 1회 더 투여한다. |
| **평가방법** | **유효성(Efficacy)**   1. 일차 유효성 평가 변수 2. Test meal 섭취 전·후 위 전체 용적의 변화 (섭취 후 15분 위 전체 용적과 섭취 전 위 전체 용적의 차이)를 측정하여 위적응 평가 3. 이차 유효성 평가 변수   1) Test meal 섭취 후 30분, 45분, 60분, 90분과 120분에서의 위배출율 |
|  | **안전성(Safety)**   1. 자⋅타각 증상 등 이상반응 2. 활력징후, 심전도, 임상실험실 검사, 체중 |
| **통계분석** | **유효성 평가변수의 분석**  유효성 평가 변수는 two-sample t-test 또는 Mann-Whitney test로 비교 검정할 것이다. 필요시 다중검정으로 인한 보정은 Bonferroni's correction으로 수행한다.  **안전성 평가변수의 분석**   - 모든 안전성 평가항목(이상반응여부, 혈액학적 검사결과 정상여부, 활력징후의 정상여부)을 임상적, 통계적으로 검토할 것이다. Chi-square test또는 Fisher’s exact test는 통계분석을 위해 이용될 것이다. |

**목 차**

**임상시험계획서 개요 2**

**용어 및 약어의 정의 7**

**임상시험 평가표(Study Flow Chart of Evaluation) 8**

**1. 임상시험의 명칭 및 단계 9**

**2. 임상시험실시기관명 및 주소 9**

**3. 시험책임자 및 시험담당자의 성명 및 직명 9**

**4. 임상시험용의약품 관리약사의 성명 및 직명 9**

**5. 임상시험의뢰자명 및 주소 9**

**6. 임상시험의 목적 및 배경 10**

**6.1. 임상시험의 목적 10**

**6.2. 임상시험의 배경 10**

**7. 임상시험용의약품의 코드명, 주성분, 원료약품 및 그 분량, 제형 등 12**

**7.1. 시험약 : Motilitone 30 mg정 12**

**7.2. 위약 : Motilitone 해당 위약 정제 12**

**7.3. 임상시험용의약품의 관리 13**

**8. 대상피험자 13**

**9. 피험자의 선정기준, 제외기준, 목표한 피험자의 수 및 그 근거 13**

**9.1. 피험자군 선정/제외기준 13**

**9.2. 목표한 피험자의 수 및 그 근거 14**

**10. 임상시험기간 15**

**11. 임상시험방법 15**

**11.1. 연구 설계 15**

**11.2. 투여약제, 투여 용법⋅용량 및 투여기간 15**

**11.3. 용량 설정의 근거 16**

**11.4. 무작위 배정 방법 16**

**11.5. 임상검사항목 및 관찰검사방법 17**

**11.6. MRI 측정방법 19**

**11.7. 병용 요법 및 주의 사항 20**

**11.8. 순응도 평가 20**

**12. 예측 부작용 및 사용상의 주의사항 15**

**13. 중지 및 탈락 기준 15**

**14. 유효성 및 안전성 평가기준 및 평가방법 15**

**14.1. 유효성 평가 변수 21**

**14.2. 안전성 평가 항목 22**

**15. 분석집단 정의 및 통계분석 방법 15**

**15.1. 분석집단 정의(Analysis set) 22**

**15.2. 통계학적 이슈(Statistical Issues) 23**

**15.3. 통계분석 방법(Statistical Method) 24**

**16. 안전성의 보고 방법 15**

**17. 자료관리 28**

**18. 피험자 보상에 대한 규약 28**

**19. 임상시험 후 피험자의 진료 및 치료 기준 28**

**20. 피험자의 안전 보호에 관한 대책 29**

**21. 기타 임상시험을 안전하고 과학적으로 실시하기 위하여 필요한 사항 30**

**22. 참고 문헌 30**

# ❖ 용어 및 약어의 정의

| 5-HT | 5-Hydroxy-Tryptamine; Serotonin |
| --- | --- |
| ADR | Adverse Drug Reaction; 이상약물반응 |
| AE | Adverse Event; 이상반응 |
| CRF | Case Report Form; 증례기록서 |
| MRI | Magnetic Resonance Imaging |
| ECG | Electrocardiogram; 심전도 |
| EPS | Epigastric Pain Syndrome; 명치부위 통증 증후군 |
| ICH | The International Conference on Harmonisation of Technical Requirements for Registration of Pharmaceuticals for Human Use |
| ITT | Intention-To-Treat |
| KGCP | Korea Good Clinical Practice |
| KGMP | Korea Good Manufacturing Practice |
| LOCF | Last Observation Carried Forward Analysis |
| NDI-K | Nepean Dyspepsia Index-Korean version |
| NOAEL | No Observed Adverse Effect Level |
| PDS | Postprandial Distress Syndrome; 식후 불편 증후군 |
| PP | Per-Protocol |
| AST | Aspartate aminotransferase |
| ALT | Alanine aminotrasferase |
| SOP | Standard Operating Procedure |
| SUSAR | Suspected Unexpected Serious Adverse Reaction; 중대하고 예상하지 못한 이상약물반응 |
|  |  |
|  |  |
|  |  |
|  |  |
|  |  |
|  |  |
|  |  |
|  |  |

# ❖ 임상시험 평가표 1. 피험자군(Study Flow Chart of Evaluation)

| 일정  항목 | Screening | Baseline | Treatment  (Evaluation) |
| --- | --- | --- | --- |
|  | Visit 1 | Visit 2^2)^ | Visit 3 |
|  | -8 주~D1 | D1 | D7 |
| 서면동의^1)^ | • |  |  |
| 선정/제외기준 | • | • |  |
| 병력 조사 | • |  |  |
| 약물력 조사 | • |  |  |
| 무작위 배정 |  | • |  |
| 임상시험용의약품 처방 |  | •^3)^ | • |
| 약물 순응도 확인^4)^ |  |  | • |
| 미복용 의약품 회수 |  |  | • |
| 활력징후 및 신체검사 | • |  | • |
| 체중 측정 | • |  | • |
| 임상실험실 검사^5)^ | • |  | • |
| 심전도(ECG) | • |  | • |
| Test meal 복용 |  |  | • |
| 위 내시경 검사^6)^ | • |  |  |
| MRI^7)^ |  |  | • |
| 병용약물 | • |  | • |
| 이상반응 모니터링 |  |  | • |

1. 서면동의는 모든 스크리닝 검사 시행 이전에 취득 되어 져야 한다.
2. 스크리닝방문 시 선정/제외 기준 모두 당일에 확인 가능한 경우 baseline 을 Visit 2에 재방문 없이 Visit 1에 시행할 수 있다.
3. Baseline 방문 다음 날부터Motilitone 180mg(1회 30mg 2정씩)군 또는 위약을 총5일간 1일3회 투여하고, D7(Visit3) Pre meal MRI 측정 직후에 약물 1회 투여한다. 단, Visit3의 MRI촬영 일정을 고려하여 실제 복용일 보다 2~3일 이전에 임상시험의약품 처방을 허용할 수 있다.
4. Visit 3 방문 전날까지 약물 순응도가 100%인 경우에만 treatment절차를 진행할 수 있다.(100%가 아닐경우는 withdrawal)
5. 검사항목: 혈액학검사, 혈액화학검사, 요검사, 임신반응검사(Visit 1, 3)
6. 최근 6개월 이내에 시행한 위 내시경 검사 결과가 없는 대상자에 한하여 시행한다.
7. MRI: Pre meal, Post meal 15min, 30min, 45min, 60min, 90min, 120min 총 7회 측정
   1. Pre-meal MRI: Test meal을 섭취 전 60min에 측정
   2. Post-meal MRI: Test meal을 섭취 후15min, 30min, 45min, 60min, 90min, 120min 측정

# 임상시험의 명칭 및 단계

건강한 성인에서 위 적응에 대한 모티리톤(Motilitone)의 효과 연구 -자기 공명 영상을 통한 평가

Efficacy of Motilitone on gastric accommodation in healthy adult patients: Evaluation using MRI method

# 임상시험실시기관명 및 주소

삼성서울병원 서울 강남구 일원동 50

# 시험책임자 및 시험담당자의 성명 및 직명

**시험책임자**

삼성서울병원 소화기내과 이풍렬 교수

**공동연구자**

삼성서울병원 소화기내과 민병훈 교수

삼성서울병원 영상의학과 최동일 교수

삼성생명과학연구소 통계지원팀 김선우 박사

# 임상시험용의약품 관리약사의 성명 및 직명

삼성서울병원 약제부 김영순, 음혜경, 임미경, 이지희, 문혜원

# 임상시험의뢰자명 및 주소

해당없음(연구자주도 임상시험)

모니터요원 : 삼성서울병원 A-CRO팀(서울 강남구 일원동 50)

Tel : 070-7014-4168 , Fax : 02-445-3793

# 임상시험의 목적 및 배경

## 임상시험의 목적

# 1차 연구 목적

건강한 성인에서 위 적응에 대한 Motilitone의 효과를 MRI를 통하여 평가하고자 한다.

#### 2차 연구 목적

1. 건강한 성인에서 위배출율에 대한 Motilitone의 효과를 MRI를 통하여 평가하고자 한다.
2. Motilitone의 안전성 평가

## 임상시험의 배경

기능성 소화불량증은 소화성 궤양이나 종양 등의 기질적 질환이 없는데도 불구하고 지속적 혹은 반복적인 postprandial distress syndrome (PDS; early sensation or postprandial fullness)과 epigastric pain syndrome (EPS; pain and discomfort or burning in the epigastrium)이 있는 경우를 말한다. 기능성 소화불량증은 위산, 위 운동 이상, 정신사회학적 요인, *Helicobacter pylori* 감염 등 여러 병태생리가 관여하지만, 최근에는 위 적응의 감소와 위의 감각능의 이상이 기능성 소화불량증의 중요한 병인으로 알려져 있다.^1), 2)^ 위 적응은 음식이 위 내로 들어오면 위 내압이 상 승하지 않도록 상부 위가 확장하는 생리작용으로 위 내용물이 소장으로 급격히 배출되거나, 식도로 역류되지 않도록 한다.^3)^ 위의 적응이 제대로 일어나지 않으면 위 감각 역치가 저하될 수 있고,^4), 5)^ 식후 불쾌감, 포만감, 상복부 불쾌감, 팽만감, 트림, 조기 만복감, 식후 상복부 통증 등의 증상을 일으킬 수 있다.^3), 6)^ 위 적응의 이상은 기능성 소화불량증 환자에서 가장 흔히 나타나는 병태생리로서,^2)^ 비아드레날린성-비콜린성 신경 (NANC; nonadrenergic noncholinergic nerve)을 통하여 일어나며, nitric oxide가 중요한 신경전달체로 작용하는 것으로 알려져 있다.^7)^

이러한 위 적응의 평가 방법으로는 gastric barostat, gastric emptying scintigraphy, Single-photon emission computed tomography (SPECT), transpyloric flow, transabdominal ultrasound 등이 다양한 장단점을 보이며 사용되어 왔다.^8)^ MRI는 위 운동과 배출을 측정할 수 있는 방법으로 알려져 있으며 최근 위의 적응을 평가하는 방법의 하나로 새롭게 제안되고 있으며 다른 방법들의 단점을 상당 부분 극복한 방법으로 평가되고 있다.^9), 10)^ 이는 환자에게 방사선 피폭을 유발하지 않는 비침습적인 방법이며 동시에 위의 구조, 운동성, 그리고 배출을 평가할 수 있고 또한 섭취된 meal, solid and liquid phase, gastric secretion, air를 감별하는데 유용하다. 여러 연구를 통하여 liquid and solid meal에 대하여 MRI와 scinigraphy사이의 위 배출 검사 결과가 일치하는 것이 확인되었으며 MRI와 gastric barostat 사이의 식후에 측정된 기시 부위에서의 위 용적이 일치하는 것을 보였다. 이 방법은 식후 위의 적응을 평가하기 위한 목적으로 위의 다양한 위치에서의 식후 용적을 측정하기 위해서 발전한 것이다.

기능성 소화 불량증에서 발생하는 위 적응 이상의 치료를 위하여 확립된 약물 치료의 방법은 아직 없는 실정이다. 적은 수의 환자를 대상으로 한 약물 치료의 연구가 시행되고 있는 중으로 nitric oxide의 역할 때문에 nitric oxide donor가 위의 이완을 강화시키고 소화불량 증상을 호전시킬 수 있다고 하며 평활근 세포 이완을 촉진시킬 수 있도록 nitric oxide-induced cGMP를 증가시키기 위한 목적으로 sidenafil 등이 연구되었으나 위의 적응 장애에 대한 효과는 불명확하였다. 선택적 serotonin 재흡수 억제제는 정상 성인에서 위의 적응을 강화시켰다고 보고된 바가 있으나 소화불량증 환자에서의 연구는 평가가 된 바가 없다. 5-HT4 receptor agonists인 cisapride 가 도움이 된다고 알려져 있었지만 현재는 심각한 부정맥 때문에 사용을 하지 못하는 현실이다.^11)^ Mosapride citrate도 널리 사용되고 있는 약제이지만 그 효과에 대하여 논란이 있다. Itopride는 전체적인 소화불량의 증상을 의미 있게 호전시킨다고 보고된 바가 있지만^12)^ 최근 유럽 3상 연구에서는 효과를 확인하지 못한 상태로 보다 현실적으로 더 효과적이면서 안전한 위장관 운동 촉진제가 필요하다. Motilitone은 Pharbitis Semen과 Corydalis Tuber로 제조된 새로운 위장 운동 촉진제로서 여러 동물 실험을 통하여 위의 배출과 위의 적응에 대한 효과가 보여진 바가 있다.^13)^

본 연구는 건강한 성인에서 위 적응에 대한 Motilitone의 효과를 MRI를 사용하여 평가하고자 한다. 위적응이란 음식물 섭취 후 gastric tone의 감소와 gastric compliance의 증가를 의미한다.

평가방법으로는 음식 섭취 전 MRI를 촬영하여 total gastric volume과 proximal gastric volume을 측정하고 음식 섭취 후 다시 MRI를 촬영하여 total gastric volume과 proximal gastric volume을 측정하여 음식 섭취 전후의 total gastric volume과 proximal gastric volume의 차이를 각각 구할 예정이다.

# 임상시험용의약품의 코드명, 주성분, 원료약품 및 그 분량, 제형 등

## 시험약 : 모티리톤(Motilitone) 30 mg정

이 약 1정중 (총 중량 약 162mg)

| 주성분 | 현호색·견우자(5:1) 50% 에탄올 연조엑스 (9.5~11.5🡪1)  (Corydaline 으로서)  (Chlorogenic acid로서) | 별 규 | 30mg  (0.156~0.468mg)  (0.111~0.333mg) |
| --- | --- | --- | --- |
| 부형제 | 규산칼슘 | 유에스피 | 6mg |
| 부형제 | 유당수화물 | 약 전 | 60mg |
| 부형제 | 미결정셀룰로오스 | 약 전 | 49.5mg |
| 붕해제 | 크로스카멜로스 나트륨 | 엔에프 | 3mg |
| 활택제 | 스테아르산 마그네슘 | 약 전 | 적량 |
| 코팅제 | 오파드라이 OY-C-7000A 흰색 | 별규 | 적량 |
| 코팅제 | 오파드라이 80W64830 분홍 | 별규 | 적량 |
| 코팅제 | 오파그로스 97W19196 클리어 | 별규 | 적량 |

- 1. **위약(Placebo)**

이 약 1정중 (총 중량 약 162mg)

| 부형제 | 유당수화물 | 약 전 | 74.2mg |
| --- | --- | --- | --- |
| 부형제 | 미결정셀룰로오스 | 약 전 | 75mg |
| 활택제 | 스테아르산마그네슘 | 약 전 | 적량 |
| 코팅제 | 오파드라이 OY-C-7000A 흰색 | 별규 | 적량 |
| 코팅제 | 오파드라이 80W64830 분홍 | 별규 | 적량 |
| 코팅제 | 오파그로스 97W19196 클리어 | 별규 | 적량 |

정제의 성상 : 분홍색의 타원형 필름코팅 정제

저장방법 : 기밀용기, 실온보관

포장 : 32정/BTL= (2정X3회X5일)+2정 [30mg/정]

## 임상시험용의약품의 관리

임상시험용의약품 관리약사(이하 ‘관리약사’)는 임상시험용의약품의 인수⋅보관⋅조제⋅관리 및 반납에 대한 책임을 갖는다.

관리약사는 임상시험용의약품의 수령사실 및 수량을 서면으로 확인하고 서명해야 하며 적절히 관리한다. 임상시험용의약품이 임상시험계획서에 따라서만 피험자에게 투여되도록 하고, 각 피험자에게 지급된 임상시험용의약품 및 관리에 대한 기록을 정확히 한다. 사용되지 않은 임상시험용의약품은 파기 또는 회수에 관해 연구자가 결정할 때까지 보관한다.

임상시험용의약품의 라벨은 아래와 같은 사항을 기재한다.

1. “임상시험용”이라는 표시
2. 제품의 코드명 또는 주성분의 일반명
3. 제조번호 및 사용(유효)기한 또는 재검사일자
4. 저장방법
5. 의약품제조업자 또는 수입자의 상호

(임상시험계획 승인을 받은 자의 상호와 주소)

1. “임상시험외의 목적으로 사용할 수 없음”이라는 표시

# 대상피험자

위장관계 증상이 없는 건강한 성인

# 피험자의 선정기준, 제외기준, 목표한 피험자의 수 및 그 근거

## 선정/제외기준

- - 1. **선정 기준**
  1. 만 20세 이상 만 70세 미만의 성인
  2. 상복부 통증 혹은 불편감에 대해 무증상인 자
  3. 본 시험에 참여할 것을 자발적으로 서면 동의한 자
  4. 최근 6개월 이내 내시경 검사상 기질적 병변이 없는 자
     1. **제외 기준**

1. 다른 기능성 위장 질환의 증상을 함께 보이는 환자
2. 임신 혹은 수유 중인 환자
3. 복부 수술의 기왕력이 있는 환자
4. 당뇨 환자
5. 심각한 다른 기저 질환이 있는 환자
6. 사용되는 약물에 알레르기가 있는 경우
7. 위장 운동 촉진제나 제산제 등을 복용하고 있는 환자는 최소 2주 전에 중단하여야 한다.
8. 간기능장애 (AST/ALT 수치가 정상 상한치의 2.5배 이상)가 있는 자
9. 신기능장애 (Serum creatinine이 정상 상한치의 1.5배 이상)가 있는 자
10. 아래와 같은 MRI검사를 받을 수 없는 환자

1) 심장박동기(Cardiac pacemaker)를 시술한 분

2) 동맥류협자(metallic aneurysm clip)를 시술한 분

3) 신경자극기(neurostimulator)를 시술한 분

4) 달팽이관 이식(cochlear implant)를 시술한 분

5) 임신중이거나 임신이 의심되는 분

1. 가임기 여성이면서 피임에 동의하지 않은 자
2. 기타 위 항목들 외에 연구자가 시험 참여에 부적합하다고 판단한 경우

## 목표한 피험자의 수 및 그 근거

- - 1. **목표 피험자 수**

총 피험자 수: 40명 (탈락율 20% 적용)

| **분류** | **건강한 피험자** | | |
| --- | --- | --- | --- |
|  | **시험군**  **(Motilitone 180mg복용군)** | **대조군**  **(위약 복용군)** | **총 수** |
| **유효성**  **평가례 수** | 16명 | 16명 | 32명 |
| **탈락율 20%**  **포함한 수** | 20명 | 20명 | 40명 |

- - 1. **피험자 수 산정 근거**

피험자 수 산정은 기존 문헌에 근거하여 (ref: Neurogastroenterol Motil. 2009; 21: 697-e37^14)^) 일차변수의 표준편차를 50 mL로 가정하고, 실험군과 대조군간 일차변수가 최소한 50 mL 이상 차이남을 기대할 때, 유의수준 5%, 검정력 80%하에서 20% 탈락율을 예상하면 다음의 공식에 의거하여 각 군당 20명이 필요하다.

n= 2*(1.96+0.84)^2^*50^2^/50^2^ (각 군당 필요한 피험자 수)

일차 변수는 “Difference between initial postprandial volume and fasting volume prior to meal administration”이다.

# 임상시험 기간

IRB/KFDA승인일~2013년 12월

# 임상시험 방법

## 연구 설계

위장관계 증상이 없는 건강한 성인을 대상으로 단일기관, 전향적, 이중눈가림, 위약대조, 무작위배정, 평행, 비교 시험을 시행한다.

**<피험자군>**

**
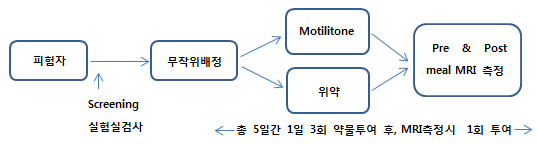
**

## 투여약제, 투여 용법⋅용량 및 투여기간

스크리닝을 통과한 피험자는 Motilitone 180mg(1회 30mg 2정씩) 또는 위약을 배정

후 Day1 아침부터 총 5일간 1일 3회 식전에 경구 투여한다.

**<피험자군>**

|  | Screening | | Baseline | | Treatment  (Evaluation) |  |  |
| --- | --- | --- | --- | --- | --- | --- | --- |
| ↓ | | ↓ | | ↓ | | | |
| V1(-8주~ D1) | | V2(D1) 무작위배정,  익일부터 총5일간 1일 3회 약물복용 | | V3(D7)  Pre & Post meal MRI측정  Pre meal MRI측정 직후  1회 약물복용 | | | |

투여약제의 종류

| 구분 | 용량 | 1회  복용 수 | 1일  복용 횟수 | 투여약제 |
| --- | --- | --- | --- | --- |
| 피험자군 | Motilitone 30 mg군 | 2정 | 3회 | Motilitone 30 mg정 |
|  | 위약군 | 2정 | 3회 | Motilitone 해당 위약정 |

##

## 용량 설정의 근거

2007년 10월부터 2008년 7월까지 진행하였던 2상 임상시험은 Motilitone 22.5 mg/day를 최저용량으로 설정하여 45, 90 mg/day까지의 용량군을 대상으로 진행하였다. 그 결과 Motilitone 은 각 치료군별 이상약물반응 발현율(90% 정확 신뢰구간)에 대한 치료군간 차이는 없었으며 선형관계가 없는 것으로 나타났다. 2009년 10월 부터 2010년 9월까지 진행된 3상임상은 Itopride를 양성대조약물로 비교평가 하였고 주유효성 평가변수인 8가지 기능성 소화불량 증상에 대한 괴로운 정도의 점수변화에 대한 투여군 간 차이의 95% 신뢰구간 상한치가 비열등임상시험 허용한계보다 작게 나타남으로 Motilitone 90mg군이 Itopride 150mg군보다 열등하지 않았다.

Motilitone 정은 현호색과 견우자(흑축)에서 추출한 생약추출물 제제이다. 따라서 기존에 시판되고 있는 유사제제는 없으므로 임상에서의 투여용량의 범위를 예측할 만한 비교제제는 없는 실정이었다. 전임상시험에서의 유효용량은 0.3~10 mg/kg이었으며 이번 연구의180mg/day용량은 단회 용량으로 1mg/kg(60kg기준)에 해당되며 이는 rat용량으로 환산하면 6.2mg/kg이므로 전임상 유효용량범위내의 용량이다.

독성시험에서는 랫드의 반수치사량은 2 g/kg, 26주 반복독성시험의 무영향량(NOAEL)은 500 mg/kg이었고, 개에서의 무영향량(NOAEL)은 100 mg/kg인 것으로 판단되었다. 1주 반복독성시험에서 NOAEL은 500mg/kg이었다. Starting dose를 구하면 다음과 같으므로 180mg/day의 용량은 매우 안전한 용량으로 판단된다.

| NOAEL | HED mg/kg÷[km_human_/km_animal_] | | Safety factor | Starting dose  (60Kg 기준) |
| --- | --- | --- | --- | --- |
| 100mg/kg in 13weeks, Dog | 100 mg÷1.8 = | 180 mg/kg | 10 | HED≒10ⅹ60= 1,080 mg |
| 500mg/kg, in  1week Dog | 500mg÷1.8= | 900 mg/kg | 10 | HED≒10ⅹ60= 5,400 mg |
| 600mg/kg in 26weeks, rat | 600mg÷6.2= | 96.72mg/kg | 10 | HED≒10ⅹ60= 580.3mg |

## 무작위 배정 방법

- - 1. **개요**

임상시험은 시험군 또는 대조군을 무작위 배정하여 병행 시험으로 진행하며, 연구에 참여한 피험자는 부여 받은 피험자 번호 순서에 따라 미리 생성된 무작위 배정표 대로 각 시험약 또는 대조약을 배정 받는다. 본 임상시험에서 각 군별 배정비율은 1:1로 한다. 피험자의 무작위 배정번호는 block size=4인 random permuted block design에 의해 작성된 무작위 배정표를 이용하여 임상시험 실시 전 선정기준 및 투약기준에 적합하고, 제외기준에 해당되지 않는 경우에 한하여 피험자가 등록되는 순서대로 부여한다. 피험자가 임상시험을 중단하면 피험자 번호를 다시 사용하지 않도록 하며, 그 피험자는 임상시험에 다시 참여할 수 없다.

- - 1. **절차**

### 눈가림 및 눈가림 해제 절차

삼성서울병원 A-CRO 의 독립된 이중맹검관리자가 무작위 배정표를 관리하되, 응급상황시 연구자의 요청에 따라 구두 혹은 서면(e-mail)등으로 맹검해제 절차를 수행할 수 있다. 단, 임상시험 진행 시 피험자에게 중대한 이상반응 등 응급상황이 발생한 경우, 연구자 및 이중맹검관리자는 피험자의 권익과 안전을 위하여 반드시 필요하다고 판단되는 경우에만 눈가림을 해제하여야 하며, 눈가림 해제가 필요한 경우 연구자는 눈가림 해제 필요성에 대한 세부사항을 반드시 시험기관 IRB 및 이중맹검관리자에게 알려야 한다.

눈가림이 유지되지 않은 피험자는 시험을 중단할 수 있으나 윤리적인 이유 등으로 피험자의 임상시험 지속이 요구되는 경우, 연구자는 이중맹검관리자와 해당 사항에 대하여 협의하여야 한다.

## 임상검사항목 및 관찰검사방법

1. Visit 1(Screening 방문: -8주 ~ 1일)

임상시험에 들어가기 전 본 임상시험의 목적과 내용에 대하여 피험자에게 상세히 설명하고, 서면으로 동의을 취득 후 피험자는 선별과정을 거칠 것이다. 선별과정은 본 임상시험의 참여 가능성 확인을 위해 아래 항목을 포함한다.

1. 서면동의
2. 인적사항 및 선정/제외기준

피험자의 성명 등 인적사항, 성별, 연령, 신장, 체중, 선정/제외기준 항목을 조사하여 기록한다.

1. 활력징후(vital signs) 및 신체검사

혈압, 맥박수 및 신체검사

1. 병력(medical history) 조사
2. 약물력 조사

시험시작 전 4주 이내에 복용했던, 현재 복용 중인 병용약물을 기재한다.

1. 임상실험실 검사

• 혈액학 검사

WBC, RBC, WBC Differential count(Neutrophil, Lymphocyte, Monocyte, Eosinophil, Basophil), Hemoglobin, Hematocrit, Platelets

• 혈액화학 검사

Total protein, albumin, Globulin, Total bilirubin, AST(SGOT), ALT(SGPT), Alkaline phosphatase, BUN, Creatinine, Uric acid, Calcium, Phosphurus, LDH, CPK, Cholesterol, Glucose, Triglyceride, y-GT, Sodium, Potassium, Chloride, Prolactin, Amylase

• 요검사

pH, Specific gravity, Protein(albumin), Glucose, Ketone, RBC(blood), Urobilinogen, Nitrite

• 임신반응검사(Urine hCG test 또는 beta hCG test)

불임수술 및 폐경이 확인된 여성을 제외한 가임기 여성은 임신반응검사를 시행한다.

1. 12-lead ECG(심전도)

기본적인 기록 이외에 automatic analysis & recording이 출력되는 항목인 ventricular rate(beats/min), PR interval(msec), QRS(msec), QT/QTc(msec)를 증례기록서에 기록한다.

⑧ 위 내시경 검사

최근 6개월 이내에 내시경 검사결과가 없을 경우에만 실시한다.

1. Visit 2(Baseline 방문: 1일)

선정/제외기준에 적합한지 재확인한다. 무작위 배정표에 따라 각 피험자에게 시험

약 및 위약을 처방한다.

1. Visit 3(Evaluation 방문: D7)
2. 약물순응도 평가
3. 활력징후 및 신체검사
4. 임상실험실 검사

• 혈액학 검사

WBC, RBC, WBC Differential count(Neutrophil, Lymphocyte, Monocyte, Eosinophil, Basophil), Hemoglobin, Hematocrit, Platelets

• 혈액화학 검사

Total protein, albumin, Globulin, Total bilirubin, AST, ALT, Alkaline phosphatase, BUN, Creatinine, Uric acid, Calcium, Phosphurus, LDH, CPK, Cholesterol, Glucose, Triglyceride, y-GT, Sodium, Potassium, Chloride, Prolactin, Amylase

• 요검사

pH, Specific gravity, Protein(albumin), Glucose, Ketone, RBC(blood), Urobilinogen, Nitrite

• 임신반응검사(Urine hCG test 또는 beta hCG test)

불임수술 및 폐경이 확인된 여성을 제외한 가임기 여성은 임신반응검사를 시행한다.

1. 12-lead ECG(심전도)

기본적인 기록 이외에 automatic analysis & recording이 출력되는 항목인 ventricular rate(beats/min), PR interval(msec), QRS(msec), QT/QTc(msec)를 증례기록서에 기록한다.

1. MRI촬영

Test meal로 뉴케어200ml(200kcal, carbohydrate: protein: fat =57: 15: 27 )를 물 200 mL와 혼합한 후 섭취하도록 한다. 섭취 전, 섭취 후 15분, 30분, 45분, 60분 90분, 120분에 (총 7회) MRI 촬영을 시행 한다.

1. 미복용 의약품 회수
2. 병용약물 조사
3. 이상반응 모니터링

피험자의 자발적인 보고 이외에 시험 담당자의 문진 등을 통하여 확인하며, 증상 출현시기, 지속시간, 이상반응의 정도 및 인과관계에 대하여 상세히 기록한다.

※ 연구과정에서 채취된 조직 검체는 삼성서울병원의 보관 관련 규정 법규에 따라 진단검사의학과에서 즉시 폐기합니다.

## MRI 측정방법

스크리닝을 통과한 피험자는 MRI를 측정한다.

Test meal로 뉴케어200ml(200kcal, carbohydrate: protein: fat =57: 15: 27 )를 물 200 mL와 혼합한 후 섭취하도록 한다. 섭취 전60분, 섭취 후 15분, 30분, 45분, 60분 90분, 120분에 (총 7회) MRI 촬영을 시행 한다.


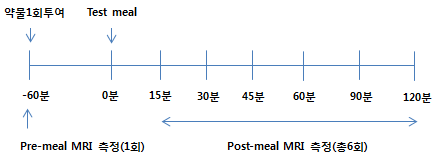


## 병용 요법 및 주의 사항

연구약물의 약효 평가에 영향을 줄 수 있는 다음의 약물은 시험기간 동안 복용이 금지된다.

☞ 병용금기약물 : 위장관운동조절제, H_2_수용체 길항제, 프로톤 펌프 저해제, 비스테로이드성 항염제, 항콜린제, 에리스로마이신, 부신피질호르몬제, 항우울제 등

시험기간 중 병용 투여한 약물이 있는 경우, 약제명, 1일 투여량, 투여기간, 투여사유 등을 증례기록서에 기재한다.

## 11.8. 순응도 평가

환자일지에 피험자가 기록한 임상시험용의약품 투여 여부를 참조하여 다음과 같은 식으로 순응도를 평가한다.

$$\text{순응도 }=\frac{\text{실제 투여 횟수}}{\text{총 투여해야 하는 횟수}}\times100$$

# 예측 부작용 및 사용상의 주의사항

1. **예측 부작용**

Motilitone 전임상 안전성 시험 결과, 임상적으로 예측되는 특별한 부작용은 거의 없었다. 기 실시했던 2상 임상시험 결과, 총 182명 중 34명(18.7%)에게서 52건의 이상약물반응이, 3상 임상시험 결과에서는 총 231명 중 20명(8.7%)에게서 31건의 이상약물반응이 발현되었으며, 일반적으로 일시적이었고 그 정도도 대부분 경증 내지 중등증이었다. 가장 흔하게 나타난 이상반응은 prolactin 상승과 설사이었다.

다음은 2,3상 임상시험에서 보고된 이상반응이다.

소화기계 : 트림, 조기만복감, 복명, 변비, 설사, 복부팽만감, 오심, 상복부통증

순환기계 : 심계항진

신경계 : 두통, 현기증

호흡기계 : 숨쉬기가 좋지 않음

피부 : 가려움증, 발진, 두드러기, 피부 동통

임상검사치 : CPK 상승, LDH 상승, prolactin 상승, eosinophil 상승, ALT 상승, Amylase 상승, GGT 상승, AST 상승

**사용상의 주의사항(일반적 주의)**

- 소아의 손에 닿지 않는 곳에 보관할 것

- 임부 및 수유부에게는 투여하지 말 것

- 파두(巴豆)와의 배합(配合)을 금함

# 중지 및 탈락 기준

1. 피험자가 임상시험책임자의 판단없이 시험약의 유효성 및 안전성을 평가하는데 영향을 줄 것으로 예상되는 약물을 투여한 경우(11.7 병용 요법 및 주의사항 참조)
2. 피험자가 임상시험용의약품의 투여 중단을 요구하는 경우
3. 중대한 이상약물반응 또는 임상검사치의 이상 변동이 나타나 시험 지속이 곤란하다고 판단되는 경우
4. 기타 연구자가 시험을 중지하여야 한다고 판단한 경우
5. 약물 순응도 평가시 100%가 안될 경우(11.8 순응도 평가식 참조)

상기 기준에 따라 중지⋅탈락한 예로부터 중지⋅탈락 시까지 얻은 시험의 결과는 최종 평가 시 평가 가능한 항목에 대해서 검토될 수 있다.

# 유효성 및 안전성 평가기준 및 평가방법

## 유효성 평가 변수

1. **1차 유효성 평가 변수:**

Test meal 섭취 전·후 위 전체 용적의 변화 (섭취 후 15분 위 전체 용적과 섭취 전 위 전체 용적의 차이)

1. **2차 유효성 평가 변수:**
   1. Test meal 섭취 후 30분, 45분, 60분, 90분과120분에서의 위배출율

## 안전성 평가 항목

1. 이상반응

이상반응이란, 약물과의 관련여부와 상관없이 시험도중 발생하는 모든 바람직하지 않은 해부, 생리학적 병변이나 대사기능의 병변으로 인해 나타나는 신체증상, 징후 및 임상실험실검사치의 변화 등을 말한다. 여기에는 기존상태의 악화, 병발질환, 약물상호작용 등도 포함된다.

발현된 모든 이상반응은 코드화를 실시하며, 각 치료군별 및 신체기관별로 분류하여 자세한 설명과 함께 빈도 및 비율을 제시한다. 각 치료군별로 임상시험용의약품과의 인과관계가 있는 이상반응과 인과관계가 없는 이상반응의 빈도를 기록한다.

1. 임상실험실 검사(이상반응 발생 예방 및 발생 시 신속한 처치를 위한 검사이므로, 2회 이상 측정시 각 시점마다 분석은 필요없음)

• 혈액학 검사

WBC, RBC, WBC Differential count(Neutrophil, Lymphocyte, Monocyte, Eosinophil, Basophil), Hemoglobin, Hematocrit, Platelets

• 혈액화학 검사

Total protein, albumin, Globulin, Total bilirubin, AST, ALT, Alkaline phosphatase, BUN, Creatinine, Uric acid, Calcium, Phosphurus, LDH, CPK, Cholesterol, Glucose, Triglyceride, y-GT, Sodium, Potassium, Chloride, Prolactin, Amylase

• 요검사

pH, Specific gravity, Protein(albumin), Glucose, Ketone, RBC(blood), Urobilinogen, Nitrite

• 임신반응검사(Urine hCG test 또는 beta hCG test)

: 검사 결과 임상적으로 의미있는 비정상 수치 여부

1. 활력징후, 체중 및 심전도

: 검사 결과의 정상여부

# 분석집단 정의 및 통계분석 방법

## 분석집단 정의(Analysis set)

### Intention-To-Treat analysis set(ITT analysis set)

무작위배정을 받은 모든 피험자 집단을 의미한다.

- - 1. **“Full Analysis set(FA set)”**

ITT analysis set’ 피험자 중 무작위배정 후 임상시험용의약품을 투여 받지 않았거나 선정제외기준에 부적합한 피험자, 임상시험용의약품 투여 후 일차유효성 평가변수에

대해 평가가 이루어지지 못한 피험자를 제외한 집단을 의미한다.

### Per Protocol analysis set(PPA set)

FA set 피험자 중 프로토콜대로 임상시험을 완료(MRI촬영 완료시점)한 피험자 집단을 의미한다. 추적조사의 실패 없이 임상시험을 종료하고, 중대한 임상시험계획서 위반사항이 없는 피험자 집단을 의미한다.

중대한 임상시험계획서 위반사항은 다음과 같다. 다음에 해당되는 피험자를 제외한 집단을 PPA set으로 규정한다..

a) 선정/제외기준 위반

b) 순응도 기준(100%)을 만족하지 못하는 경우

c) 무작위배정 위반

d) 병용금지약물의 복용

e) 기타 중대한 임상시험계획서 위반사항이 발생한 경우

### Safety population(Safety set)

“Safety population(이하 Safety set)”은 무작위배정 후 임상시험용의약품을 한번이라도 복용하고, 이후 안전성 관련 follow-up이 이루어진 피험자 집단을 의미한다.

### 유효성 및 안전성 분석 대상 피험자

본 시험의 피험자로부터 얻어진 자료는 FA set와 PPA set의 2가지 형태로 분석된다. 유효성에 대한 결과는 원칙적으로 FA set를 분석한 결과를 주분석 결과로 하고, PPA set를 대상으로 분석한 결과는 추가적결과로 간주한다. 안전성에 대한 자료는 Safety set을 대상으로 실제 투여 받은 대로 분석한다.

## 통계학적 이슈(Statistical Issues)

### 결측치 보정 방법(Handling of Missing Data)

약물 투여 후 1회 방문이 이루어지므로, 약물 투여 후 발생한 결측치에 대해서는 보정을 하지 않는다. (순응도 100%인 연구 참여자만 분석에 포함)

### 중간분석(Interim Analysis)

계획되지 않음.

### 일반적 통계 원칙(General Statistical Issues)

1. 모든 통계분석은 SAS(Ver. 9.1, SAS Institute, Cary, NC, USA)를 사용한다.
2. 모든 통계검정은 유의수준 5%하에서 양측검정(two-sided)을 원칙으로 한다.
3. PPA set와 Safety set 분석은 결측치 보정 없이 관측치 그대로 사용한다.

### 다중비교(Multiple Comparison)

다중비교 필요시 다중비교검정법을 사용한다.

- - 1. **다중검정 (Multiple testing)**

다중검정 필요시 Bonferroni's correction으로 위양성율의 증가를 보정한다.

## 통계분석 방법(Statistical Method)

### 인구통계학적 정보 및 투여 전 특성 (Demography and Baseline Characteristics)

임상시험에 등록되어 무작위배정 받은 모든 피험자에 대한 인구통계학적 정보(연령, 성별 등)와 시험전 면담(음주여부, 흡연여부, 소화불량증 병력기간 등)에 대해서 연속형 자료는 기술통계량(피험자 수, 평균, 표준편차, 중앙값, 최소값, 최대값)을 제시하고, 범주형 자료는 빈도와 비율을 제시한다. 투여군 간 비교를 위해서 연속형 자료에 대해서는 t-검정(two sample t-test) 또는 윌콕슨 순위합 검정(Wilcoxon rank sum test)을 사용하여 유의성을 검토하고, 범주형 자료에 대해서는 카이제곱 검정(chi-square test) 또는 피셔의 정확 검정(Fisher’s exact test)을 사용하여 유의성을 검토한다.

인구통계학적 정보 및 투여 전 특성의 비교에서 군간 유의한 차이를 보이는 항목은 주유효성 변수에 미치는 영향 여부에 따라 유효성 분석 시 보정하여 분석할 수 있다.

### 유효성 평가 변수의 분석 (Statistical Analysis of Efficacy data)

(1) 일차 유효성 평가 변수

- 각 군별 Test meal 섭취 전, 섭취 후, 섭취 전 후 위 전체 용적의 변화량의 기술통계는 평균, 중위수, 표준편차, 사분위범위 등으로 제시한다.

- Test meal 섭취 전 후 위 전체 용적의 변화량의 두 군간 차이는 정규성 여부에 따라 two-sample t-test 또는 Mann-Whitney test로 비교 검정한다. 만약 두 군간 차이가 나고 변화량과 연관이 있는 변수가 있다면 이 변수를 covariate으로 보정하는 다중회귀분석 (정규성 여부에 따라 변수 변환 후 수행 가능) 또는 비모수 편상관분석 (정규성을 맞추지 못할 경우)을 실시하여 두 군간 차이를 검정한다.

(2) 이차 유효성 평가변수

- 각 군별 시점 (섭취 후 30분, 45분, 60분, 90분, 120분)에서 위배출율에 대한 기술통계는 평균, 중위수, 표준편차, 사분위범위 등으로 제시한다.

- 각 군별 시점별 위배출율의 두 군간 차이는 정규성 여부에 따라 two-sample t-test 또는 Mann-Whitney test로 비교 검정하되, 다중검정으로 인한 false positive error의 크기 증가 보정을 위해 Bonferroni's correction을 수행한다. 만약 두 군간 차이가 나고 위배출율과 연관이 있는 변수가 있다면 이 변수를 covariate으로 보정하는 다중회귀분석 (정규성 여부에 따라 변수 변환 후 수행 가능) 또는 비모수 편상관분석 (정규성을 맞추지 못할 경우)을 실시하여 두 군간 차이를 검정한다. 이 경우에도 마찬가지로 Bonferroni's correction이 추가된다..

### 안전성 평가 변수의 분석(Statistical Analysis of Safety data)

안전성 평가변수의 기술통계는 빈도, 비율 및 비율의 95% 신뢰구간으로 제시하며 Chi-square test 또는 Fisher’s exact test로 비교 검정한다.

# 안전성의 보고 방법

1. **이상반응의 정의**

이상반응(Adverse Event, AE)은 임상시험용의약품을 투여받은 피험자에서 발생한, 바람직하지 않고 의도되지 않은 징후(Sign), 증상(Symptom), 질병을 말하며, 해당 임상시험용의약품과 반드시 인과관계를 가져야 하는 것은 아니다. 이때 임상시험용의약품과의 인과관계를 배제할 수 없는 경우는 이상약물반응(Adverse Drug Reaction, ADR)이라 한다.

1. **중대한 이상반응/이상약물반응의 정의**

중대한 이상반응/이상약물반응(Serious AE/ADR)은 임상시험용의약품의 임의의 용량에서 발생한 이상반응 또는 이상약물반응 중에서 다음 각목의 1에 해당하는 경우를 말한다.

1. 사망을 초래하거나 생명을 위협하는 경우
2. 입원 또는 입원 기간의 연장이 필요한 경우
3. 지속적 또는 의미있는 불구나 기능 저하를 초래하는 경우
4. 선천적 기형 또는 이상을 초래하는 경우
5. 기타 의학적으로 중요한 상황
6. **이상반응의 기록**

임상시험 중 발생하는 모든 이상반응을 기록하는 것은 임상시험책임자와 담당자의 의무이다. 이상반응은 의학 진단 용어로 기록하여야 하며, 이것이 불가능할 경우 임상시험책임자 또는 담당자가 관찰하거나, 피험자가 보고한 증상 및 징후에 대한 용어를 기록한다.

임상시험 중에 발생한 이상반응을 조사하여 증례기록양식의 “이상반응” 양식에 증상 및 징후, 증상의 발현기간(발현 및 소실 날짜), 중증도(경증, 중등증, 중증), 중대한 이상반응의 여부(예/아니오), 임상시험용의약품과의 인과관계, 이상반응에 관련하여 취해진 조치, 이상반응의 진행상황 등에 관하여 기록한다.

또한, 임상검사치의 경우 임상적으로 의미있는 비정상치를 파악하여 증례기록서에 기록한다.

1. **이상반응의 중증도**

| 1 | 경증(Mild) | 피험자가 거의 느끼지 못할 정도로 정상적인 일상생활(기능)을 저해치 않는 정도. 대부분 치료가 필요하지 않는 정도 |
| --- | --- | --- |
| 2 | 중등증(Moderate) | 피험자가 불편감을 느낄 수 있으며, 정상적인 일상생활(기능)을 저해하는 정도. 피험자가 시험을 계속 할 수는 있으나 치료가 필요할 수도 있는 정도. |
| 3 | 중증(Severe) | 피험자가 매우 불편하여 일상생활(기능)이 불가능하고, 시험의 계속적인 참여가 불가능한 정도. 치료나 입원이 필요할 수 있는 정도 |

1. **임상시험용의약품과의 인과관계**

| 1 | 관련성이 명백함  (Definitely) | - 이 약을 투여하였다는 증거가 있는 경우 - 이 약 투여와 이상반응 발현의 시간적 순서가 타당한 경우 - 이상반응이 다른 어떤 이유보다 이 약 투여에 의해 가장 개연성있게 설명되는 경우 - 투여중단으로 이상반응이 사라지는 경우 - 재투여(rechallenge, 가능한 경우에만 실시) 결과가 양성인 경우 - 이상반응이 이 약 또는 동일계열의 약물에 대해 이미 알려져 있는 정보와 일관된 양상을 보이는 경우 |
| --- | --- | --- |
| 2 | 관련성이 많음  (Probably) | - 이 약을 투여하였다는 증거가 있는 경우 - 이 약 투여와 이상반응 발현의 시간적 순서가 타당한 경우 - 이상반응이 다른 원인보다 이 약 투여에 의해 더욱 개연성 있게 설명되는 경우 - 투여중단으로 이상반응이 사라지는 경우 |
| 3 | 관련성이 의심됨  (Possibly) | - 이 약을 투여하였다는 증거가 있는 경우 - 이 약 투여와 이상반응 발현의 시간적 순서가 타당한 경우 - 이상반응이 다른 가능성 있는 원인들과 같은 수준으로 이 약에 기인한다고 판단되는 경우 - 투여중단으로 (실시된 경우) 이상반응이 사라지는 경우 |
| 4 | 관련성이 적음  (Probably not) | - 이 약을 투여하였다는 증거가 있는 경우 - 이상반응에 대해 보다 가능성 있는 다른 원인이 있는 경우 - 투여중단 (실시된 경우) 하여도 이상반응이 사라지지 않거나 판단이 모호한 경우 - 재투여 (실시된 경우) 결과가 음성이거나 판단이 모호한 경우 |
| 5 | 관련성이 없음  (Definitely not) | - 이 약을 투여하지 않은 경우 또는 이 약 투여와 이상반응 발현의 시간적 순서가 타당하지 않은 경우 또는 이상반응의 명백한 다른 원인이 있는 경우 |
| 6 | 평가불가능  (Unassessable) | - 정보가 불충분하거나 상충되어 판단할 수 없고 이를 보완하거나 확인할 수 없는 경우 |

1. **이상반응과 관련하여 취해진 조치**

| 0 | 취해진 조치 없음(No action taken) |
| --- | --- |
| 1 | 임상시험용의약품의 투여용량 변경/일시적 중단(Study drug dosage adjusted/temporarily interrupted) |
| 2 | 임상시험용의약품의 투여 중단(Study drug permanently discontinued due to this adverse event) |
| 3 | 치료약물 병용 투여(Concomitant medication taken) |
| 4 | 비약물 치료(Non-drug therapy given) |
| 5 | 입원/입원 기간의 연장(Hospitalization/prolonged hospitalization) |

1. **중대한 이상반응/이상약물반응의 보고**

본 시험 기간 중 임상시험책임자 및 담당자는 환자의 안전에 만전을 기해야 하며 중대한 이상약물반응 발생시에는 시험을 중지하고 신속하고 적절한 조치를 취하여 이상반응을 최소화하여야 한다.

임상시험 중 “중대한 이상약물반응” 발생 시 각 담당자의 의무는 다음과 같다.

1. **임상시험자의 의무**

임상시험자는 임상시험 중 중대한 이상반응이 발생한 때는 즉시 임상시험심사위원회 및 삼성서울병원A-CRO에 보고해야 한다. 특히 중대하고 예상하지 못한 이상약물반응(SUSAR)인 경우 별도의 지시가 있을 때까지 해당 임상시험용의약품에 대한 임상시험의 일부 또는 전부를 중지하여야 한다.

시험자는 임상시험용의약품과의 관련성 유무에 관계없이 SAE를 시험자가 알게 된 시점에서 **72시간 이내**에 해당 보고양식을 이용하여 IRB및 모니터**(삼성서울병원 A-CRO팀, tel: 070-7014-4168, Fax:02-445-3793)**에게 보고하여야 한다. 필요한 경우, 시험자는 SAE에 대한 새로운 정보를 포함하는 추적조사 보고서를 작성하여 IRB와 모니터에게 보고한다.

이 경우 피험자의 신원을 보호하기 위하여 피험자의 성명, 주민등록번호 및 주소를 기재하는 대신 피험자식별코드를 사용하여야 하며, 중대한 이상반응/이상약물반응의 보고에 관한 관련지침이 있는 경우 시험자는 이에 따라야 한다.

가능하다면, 사망 예를 보고하는 경우 시험책임자는 IRB와 삼성서울병원 A-CRO팀에 부검보고서(부검을 실시한 경우에 한함)와 사망진단서 등의 추가정보를 제공한다. 또한, 중대하고 예상하지 못한 이상약물반응인 경우 후원기관(동아제약주식회사) 이태호 연구원(Tel. 031-280-1374)에게 발생 후 5일 이내에 문서로 상세한 내용이 포함된 추가 보고를 하여야 한다.

1. 임상시험심사위원회의 의무

임상시험심사위원회는 중대하고 예상하지 못한 이상약물반응(SUSAR)이 나타난 경우에는 임상시험의 일부 또는 전부에 대하여 중지명령 등 필요한 조치를 임상시험책임자에게 하여야 한다.

1. **삼성서울병원 A-CRO(모니터요원)의 의무**

시험조정자/모니터요원(삼성서울병원 A-CRO)는 기타 관련된 식품의약품안전청장과 후원기관(동아제약)에게 중대하고 예상하지 못한 이상약물반응(SUSAR)을 관련 규정에 따라 정한 기간 내에 신속히 보고해야 한다. 즉,

• 사망을 초래하거나 생명을 위협하는 경우에는 삼성서울병원 A-CRO에서 이 사실을 보고받거나 알게된 날로부터 7일 이내. 다만, 이 경우 상세한 정보를 최초 보고일로부터 8일 이내에 추가로 보고

• 다른 모든 중대하고 예상하지 못한 이상약물반응의 경우에는 삼성서울병원 A-CRO에서 이 사실을 보고받거나 알게된 날로부터 15일 이내

모니터요원은 식품의약품안전청장에게 중대하고 예상하지 못한 이상약물반응을 보고할 경우, 이상약물반응 보고서에 임상시험책임자 또는 임상시험담당자로부터 보고받은 내용을 첨부하여 보고한다. 또한 해당 중대하고 예상하지 못한 이상약물반응이 종결(해당 이상약물반응의 소실 또는 추적조사의 불가 등)될 때까지 추가적인 안전성 정보를 주기적으로 보고한다.

# 자료관리

임상시험에서 얻어진 자료의 질적 수준을 확보하기 위한 자료관리는 KGCP 규정 및 ICH 지침에 근거한 표준작업지침서(Standard Operating Procedure, SOP)에 의해 실시하며, 임상자료의 질적 보증(data quality assurance)을 위한 자료관리 과정은 임상시험 관련자(시험책임자, 시험담당자 등)에 대한 교육, 임상시험 모니터요원에 의한 모니터링 및 점검(monitoring and audit), 데이터 관리(data management) 등의 형태로 실시한다.

삼성서울병원A-CRO는 임상시험실시기관에서 임상 자료(clinical data)를 기록하기 위한 CRF를 제공하고, 시험책임자(or 시험 담당자 등)는 피험자의 정보 및 임상 검사 결과 등 피험자 자료가 발생한 시점에서 즉시 증례기록서(Case Report Form, 이하 CRF)에 기록한다. CRF는 결측사항이 발생하지 않도록 기록하며 결측자료가 발생한 경우에 대해서는 적절한 사유를 기록한다. 또한 CRF 상의 모든 수정사항에 대해서는 수정 전 결과가 보이도록 한 줄을 그어 표시한 후 수정 후 결과, 수정서명, 수정일을 기록하도록 한다(필요한 경우 수정사유 기록). 삼성서울병원A-CRO의 CRA(or Study Manager)는 CRF의 임상시험 관련 기록에 대해 Source Document Verification(SDV)를 실시하여 임상자료의 임상시험계획서 준수 여부와 정확성을 검토한다. MRI검사 기록지는 DM팀으로 바로 전달된다.

CRF에 기록된 임상자료의 입력 및 관리를 위해 데이터 관리계획(Data Management Plan, 이하 DMP)을 정의하여 데이터의 완전성, 정확성 및 일치성을 점검한다. 임상시험 자료 입력의 정확성을 보장하고, 이상반응 및 병용약물은 코딩사전(Coding Dictionary)을 이용하여 코드화한다. 임상자료의 입력이 완료되면, 데이터 점검 과정(Data validation) 및 Data Quality Control(QC) 과정을 실시하여 데이터의 완결성, 정확성 및 신뢰성을 보장할 수 있도록 하고, 이 후 데이터베이스 잠금(Database Lock)을 실시한다. 맹검 해제(Code Open)를 실시하기 전에 Blind Meeting을 실시하여 객관성을 유지한다. 임상시험 데이터 관리를 위해 사용된 데이터베이스(database) 및 데이터 관리 과정에서 발생되는 모든 문서들은 시스템 오류 또는 재난(system error or environmental disasters) 등으로 인한 데이터 손실을 방지하기 위해 정기적인 백업을 실시하여 복구가 가능하도록 관리한다.

# 피험자 보상에 대한 규약

본 임상시험과 관련된 손상이 발생하여 응급조치가 필요할 경우, 삼성서울병원에서 행해지는 처음 24시간 동안의 응급조치에 필요한 치료비에 대해서 연구과제당 3회에 한하여 삼성서울병원에서 부담합니다.

# 임상시험 후 피험자의 진료 및 치료 기준

처치를 요하는 이상반응이 발생한 경우에는 전문의의 진단 및 적절한 처치를 받도록 한다.

# 피험자의 안전 보호에 관한 대책

1. 치료 전 검사를 통하여 피험자가 본 임상시험에 적절한 지를 엄격히 평가한다.
2. 임상시험계획서에 따라 임상시험을 실시하고 시험기간 중 정기적인 검사와 검진을 통하여 이상반응 및 이상약물반응의 출현 여부와 그 정도를 평가하고 적절한 조치를 취한다.
3. 임상시험실시기관은 본 시험계획서에 규정된 대로 임상시험이 적절히 진행될 수 있도록 임상시험에 필요한 설비와 전문인력을 갖추고 피험자의 안전보호에 만전을 기해야 한다.

# 기타 임상시험을 안전하고 과학적으로 실시하기 위하여 필요한 사항

1. **임상시험관리기준(KGCP)**

본 임상시험을 실시함에 있어 KGCP 및 Helsinki 선언의 근본 정신을 준수하여 윤리적이고 과학적인 배려 하에 연구를 실시하도록 한다.

1. **피험자 동의**

공고 등을 통하여 모집, 임상시험에 참여하는 피험자에게 임상시험담당자는 사전에 시험의 성격, 범위, 본 시험의 내용 및 임상시험용의약품의 효과, 이상반응, 예상되는 결과 등에 대해 사전에 쉽게 이해할 수 있도록 충분히 설명한 후, 환자의 동의를 얻어 동의서에는 임상시험담당자 및 피험자가 서명하고 증례기록양식에 동의 취득년월일(서명일)을 기재한다.

1. **환자신분기밀 보장**

피험자 기록은 피험자의 코드 번호와 이니셜에 의해서만 확인 가능하여야 하지만 정부기관의 요청이나 임상시험모니터요원의 요청 등 경우에 따라서는 임상시험책임자가 임상시험과 관계되는 임상기록이나 복사본 등을 제공할 수 있다. 서명을 받은 피험자 동의서는 시험책임자가 보관한다. 시험책임자는 피험자번호 및 피험자명이 기록된 리스트를 갖추어 놓아 나중에 기록을 찾을 수 있도록 한다.

1. **점검/실태조사**

삼성서울병원 기관장은 신뢰성 보증의 일환으로 점검(Audit)을 실시할 수 있으며, 임상시험의 실시가 계획서, 표준작업지침서 및 관련 규정에 따라 이루어졌는지를 확인할 수 있다. 또한, 식품의약품안전청장은 실태조사(Inspection)에 의해 임상시험의 실시가 임상시험관리기준 및 관련 규정에 따라 실시되었는지를 확인할 수 있다.

1. **임상시험 모니터링**

임상시험이 KGCP에 따라 실시되도록 삼성서울병원A-CRO측에서 모니터링을 실시한다. 모니터링 시에는 증례기록이 완전하고 명확한지 확인하고 임상시험자의 입회 하에 대조 검토가 필요하며 임상시험자는 임상시험모니터요원에게 협조해야 한다.

1. **참고 문헌**

**1) Mearin F, Cucala M, Azpiroz F, Malagelada JR. The origin of symptoms on the brain-gut axis in functional dyspepsia. Gastroenterology 1991;101:999-1006.**

**2) Thumshirn M, Camilleri M, Saslow SB, Williams DE, Burton DD, Hanson RB. Gastric accommodation in non-ulcer dyspepsia and the roles of Helicobacter pylori infection and vagal function. Gut 1999;44:55-64.**

**3) Tack J, Piessevaux H, Coulie B, Caenepeel P, Janssens J. Role of impaired gastric accommodation to a meal in functional dyspepsia. Gastroenterology 1998;115:1346-52.**

**4) Coffin B, Azpiroz F, Guarner F, Malagelada JR. Selective gastric hypersensitivity and reflex hyporeactivity in functional dyspepsia. Gastroenterology 1994;107:1345-51.**

**5) Distrutti E, Azpiroz F, Soldevilla A, Malagelada JR. Gastric wall tension determines perception of gastric distention. Gastroenterology 1999;116:1035-42.**

**6) Talley NJ, Silverstein MD, Agreus L, Nyren O, Sonnenberg A, Holtmann G. AGA technical review: evaluation of dyspepsia. American Gastroenterological Association. Gastroenterology 1998;114:582-95.**

**7) Desai KM, Sessa WC, Vane JR. Involvement of nitric oxide in the reflex relaxation of the stomach to accommodate food or fluid. Nature 1991;351:477-9.**

**8) Schwizer W, Steingotter A, Fox M, Zur T, Thumshirn M, Bosiger P, Fried M. Non-invasive measurement of gastric accommodation in humans. Gut 2002;51 Suppl 1:i59-62.**

**9) Schwizer W, Maecke H, Fried M. Measurement of gastric emptying by magnetic resonance imaging in humans. Gastroenterology 1992;103:369-76.**

**10) Feinle C, Kunz P, Boesiger P, Fried M, Schwizer W. Scintigraphic validation of a magnetic resonance imaging method to study gastric emptying of a solid meal in humans. Gut 1999;44:106-11.**

**11) Tack J, Broeckaert D, Coulie B, Janssens J. The influence of cisapride on gastric tone and the perception of gastric distension. Aliment Pharmacol Ther 1998;12:761-6.**

**12) Holtmann G, Talley NJ, Liebregts T, Adam B, Parow C. A placebo-controlled trial of itopride in functional dyspepsia. N Engl J Med 2006;354:832-40.**

**13) Lee TH, Choi JJ, Kim DH, Choi S, Lee KR, Son M, Jin M. Gastroprokinetic effects of DA-9701, a new prokinetic agent formulated with Pharbitis Semen and Corydalis Tuber. Phytomedicine 2008;15:836-43.**

**14) Fruehauf H, Steingoetter A, Fox MR, Kwiatek MA, Boesiger P, Schwizer W, Fried M, Thumshirn M, Goetze O. Charaterization of gastric volume responses and liquid emptying in functional dyspesia and healthy by MRI or barostat and simultaneous ^13^ C-acetate breast test. Neurogastroenterol Motil 2009;21:697-e37.**

**15) Ingrid MZ, Jeoffrey JLH, Paul V, Paul HC, Albert R, Ad AM, Gastric accommodation and motility are influenced by the barostat device: assessment with magnetic reasonace imaging, AJ Physiol Gastroinest Liver Physiol, 2007; 292: G208-G214.**
