# Supplementary material for: Effect of DA-9701 on Gastric Motor Function Assessed by Magnetic Resonance Imaging in Healthy Volunteers: A Randomized, Double-Blind, Placebo-Controlled Trial
Source: PLoS One. 2015 Sep 24;10(9):e0138927. doi: 10.1371/journal.pone.0138927 (PMC4581730; doi:10.1371/journal.pone.0138927)
Supplement: S2 Protocol — (DOCX) [file pone.0138927.s005.docx]

Clinical Study Protocol

| **Study Title :** | **Efficacy of Motilitone on gastric accommodation in healthy adult patients: Evaluation using MRI method** |
| --- | --- |
| **Protocol ID :** | **Motilitone** |
| **Protocol No. :** | **(Version 1.2)** |
|  |  |

| **Study Title** | Efficacy of Motilitone on gastric accommodation in healthy adult patients: Evaluation using MRI method |
| --- | --- |
| **Aim** | To evaluate effects of motilitone on gastric accommodation and emptying after a meal in a group of healthy volunteers using 3-D gastric volume measurements by MRI |
| **Design** | randomized, double-blind, parallel group, placebo-controlled trial |
| **Sample sizes** | Sample sizes were determined prospectively with reference to a previous study that used similar end points (ref: Neurogastroenterol Motil. 2009; 21: 697-e37^)^). Sixteen subjects per group were required for a power of 80% under the two-sided significance of 5% to detect a between-group difference of 50 mL and assuming SD of 50 mL of change in TGV after the test meal. To ensure inclusion of at least 16 subjects per group, 20 subjects per group were ultimately recruited to account for a potential withdrawal rate of 20%. |
| **Subjects** | Healthy adults |
| **Criteria** | **<Inclusion criteria>**  Healthy volunteers between 20 and 70 years of age without upper abdominal pain or discomfort and a structural abnormality on upper GI endoscopy performed within the preceding 6 months were eligible for the trial.  **<Exclusion criteria>**  (1) any functional GI disease or previous abdominal surgery  (2) diabetes mellitus under insulin or oral anti-hyperglycemic agent treatment  (3) significant cardiopulmonary diseases or any malignancies  (4) significant renal (serum creatinine level ≥ 1.5 × the upper normal limit) or liver disease (serum aspartate aminotransferase and alanine aminotransferase levels ≥ 2.5 × the upper normal limits  (5) taking medications that may alter gastric function within 2 weeks prior to the start of the study  (6) pregnancy or lactation  (7) females with inadequate contraception during the study period; (8) contraindications to MRI (e.g., cardiac pacemaker or metallic aneurysm clip)  (9) allergic history to motilitone  (10) other conditions likely to interfere with study procedures, as judged by the investigator. |
| **Study drugs** | either 60 mg (2 pills) of motilitone or placebo three times a day |
| **MRI protocol** | At the end of the study (day 7), subjects underwent gastric MRI (pre-meal MRI) and took the study drug 60 min before administration of the test meal. Post-meal MRI was performed at 15, 30, 45, 60, 90, and 120 min after completing the test meal defined as time 0 min. The test meal consisted of 200 mL Nucare® (200 kcal, carbohydrate : protein : fat = 57 : 15 : 27; Daesang, Seoul, Korea) and 200 mL water. |
| **Medication** | either 60 mg (2 pills) of motilitone (motilitone group) or placebo (placebo group) three times a day (before meals) for 5 days (days 2-6) |
| **Assessment** | **Efficacy**  1. The primary outcome  - change in total gastric volume (TGV) after the test meal (defined as difference between TGV 15 min after the test meal and at the pre-test meal)  2. The secondary outcomes  Gasatric emptying (GE) rate (%)  - change in proximal TGV after the test meal  - change in proximal to distal TGV ratio after the test meal (proximal to distal TGV ratio was calculated as proximal TGV divided by the distal TGV)  - gastric emptying rates (calculated as follows: (gastric content volume (GCV) 15 min after the test meal-GCV at 30, 45, 60, 90, and 120 min after the test meal)/GCV 15 min after the test meal x 100 (%)) |
|  | **Safety**  Self-reported adverse effects  Laboratory tests including EKG |
| **Statistical analysis** | The efficacy analysis was performed based on the modified intention-to-treat principle, which included all subjects who underwent randomization and who had endpoints that could be evaluated.  Demographics in the two groups were compared using Fisher’s exact test for categorical data and the two-sample t-test for continuous data.  Gastric emptying rate was compared by repeated measures analyses with mixed model. Analysis of covariance model was used with the corresponding baseline value (values of measurement before the test meal) as the covariate to asses changes in TGV, proximal TGV and proximal to distal TGV ratio after the test meal to increase precision of the comparisons between the two groups by accounting for variation. |
